# GSK3 suppression upregulates β-catenin and c-Myc to abrogate KRas-dependent tumors

**DOI:** 10.1038/s41467-018-07644-6

**Published:** 2018-12-04

**Authors:** Aslamuzzaman Kazi, Shengyan Xiang, Hua Yang, Daniel Delitto, José Trevino, Rays H. Y. Jiang, Muhammad Ayaz, Harshani R. Lawrence, Perry Kennedy, Saïd M. Sebti

**Affiliations:** 1 Drug Discovery Department, H. Lee Moffitt Cancer Center and Research Institute, Tampa, FL 33612 USA; 2 Department of Surgery, University of Florida, Gainesville, FL 32610 USA; 3 Department of Global Health and Center for Drug Discovery and Innovation, University of South Florida, Tampa, FL 33612 USA; 4 Chemical Biology and Molecular Medicine Program, H. Lee Moffitt Cancer Center and Research Institute, Tampa, FL 33612 USA

## Abstract

Mutant KRas is a significant driver of human oncogenesis and confers resistance to therapy, underscoring the need to develop approaches that disable mutant KRas-driven tumors. Because targeting KRas directly has proven difficult, identifying vulnerabilities specific for mutant KRas tumors is an important alternative approach. Here we show that glycogen synthase kinase 3 (GSK3) is required for the in vitro and in vivo growth and survival of human mutant KRas-dependent tumors but is dispensable for mutant KRas-independent tumors. Further, inhibiting phosphorylation of GSK3 substrates c-Myc on T58 and β-catenin on S33/S37/T41 and their subsequent upregulation contribute to the antitumor activity of GSK3 inhibition. Importantly, GSK3 blockade inhibits the in vivo growth of G12D, G12V, and G12C mutant KRas primary and metastatic patient-derived xenografts from pancreatic cancer patients who progressed on chemo- and radiation therapies. This discovery opens new avenues to target mutant KRas-dependent cancers.

## Abstract (web summary)

Direct targeting of mutant KRas is challenging and alternative approaches are needed. Here they show glycogen synthase kinase 3 (GSK3) to be required for the growth and survival of human mutant KRas-dependent tumors but dispensable for mutant KRas-independent tumors and show GSK3 inhibition to inhibit in vivo growth of Kras mutant patient-derived pancreatic tumors.

## Introduction

Ras proteins are low-molecular-weight GTP/GDP-binding GTPases that, under physiological conditions, regulate several important cellular processes, including cell growth, differentiation, and survival^1,2^. There are three human *Ras* genes that encode HRas, NRas, and KRas proteins. Ras proteins mediate the transfer of biological information from cell surface receptors to intracellular signaling pathways such as the Raf/Mek/Erk, PI3K/Akt, RalGDS/Ral, TIAM1/Rac, and p190/Rho pathways, eventually leading to regulation of gene expression, cell cycle progression, survival, cytoskeletal changes, and motility^2^.

Since their identification in mammalian cells in 1981, *Ras* genes have been shown to play pivotal roles in human tumor pathogenesis, contributing to several hallmarks of human cancer and driving tumorigenesis in genetically engineered mouse models^1,2^. Clinically, *KRas*, the most frequently mutated *Ras* gene, confers resistance to therapy in cancers such as pancreatic, colon, and lung^1,2^. Notably, patients with mutant KRas cancers have poor prognosis, increased tumor aggressiveness and metastasis, and are less likely to respond to chemotherapy and targeted therapies^3–6^, leading the National Comprehensive Cancer Network to recommend treatment with epidermal growth factor (EGF) receptor inhibitors only in patients whose tumors harbor wild-type KRas^4^. These observations prompted many to target mutant KRas, which unfortunately has proven to be difficult. Although recent efforts to understand the conformational changes and dynamics of KRas resulted in the identification of  covalent as well as non-covalent binders of KRas^7–9^, currently there are no approved therapies that directly target mutant KRas^10^. However, mutant KRas-driven cancers may gain dependencies through other pathways^11^. Here, by exploring vulnerabilities of human tumors that depend on mutant KRas, we sought to identify kinases and their corresponding pathways that mutant KRas depends on to induce malignant transformation and to target such pathways for cancer therapy.

## Results

### GSK3 is required for survival of KRas-dependent tumors

**Fig. 1:**
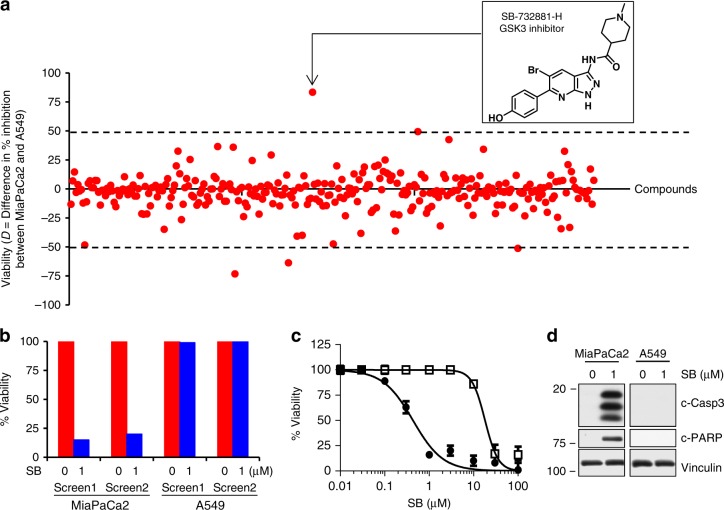
Kinome screen identifies GSK3 inhibitor that suppresses mutant KRas-dependent cells. **a** MiaPaCa2 (mutant KRas-dependent) and A549 (mutant KRas-independent) human tumor cells were treated for 72 h in 96-well plates with 304 kinase inhibitors (1 µM) using a one well-one inhibitor format. *D* [(% inhibition of viability of MiaPaCa2) − (% inhibition of viability of A549)] was determined for each compound based on the average of two screens. **b** Effects of SB on percent cell viability from both screens. **c** IC_50_ determination of in-house synthesized SB (experiment done three times). **d** Western blots showing SB-induced caspase-3 activation and PARP cleavage in MiaPaCa2 but not A549 cells (experiment done three times)

**Fig. 2:**
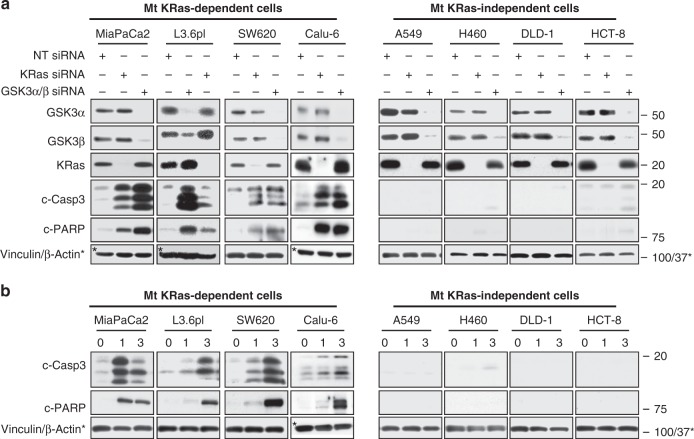
Silencing of GSK3α/β induces apoptosis only in mutant (Mt) KRas-dependent cancer cells. Mutant KRas-dependent (MiaPaCa2, L3.6pl, SW620, and Calu-6) and mutant KRas-independent (A549, H460, DLD-1, and HCT-8) human cancer cell lines were **a** transiently transfected with SMARTpool KRas, GSK3α/β, or NT siRNAs or **b** treated with the GSK3α/β inhibitor SB and processed 72 h later for western blotting for KRas, GSK3α, GSK3β, c-caspase-3, c-PARP, and Vinculin. For **a** and **b**, experiments were repeated at least three times for each cell line

**Fig. 3:**
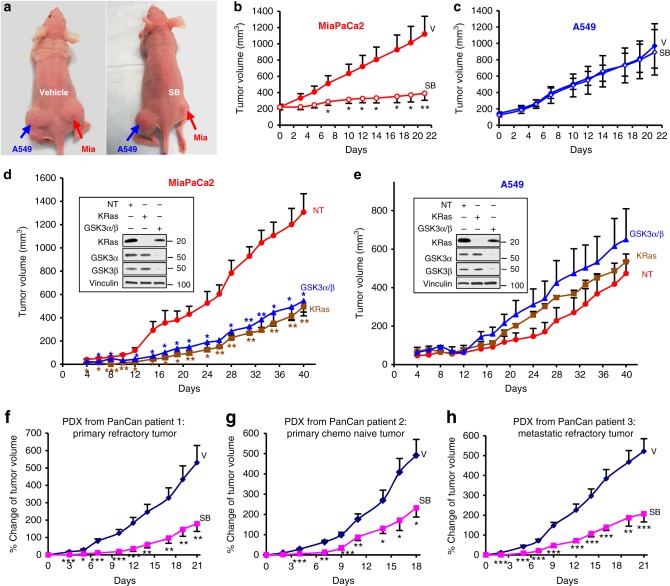
SB suppresses mutant  KRas-dependent tumors and pancreatic cancer patient xenografts. MiaPaCa2 and A549 cells were implanted on the right and left flank, respectively, and mice were treated daily with vehicle (V) or SB (50 mg/kg). **a** Representative mice treated with either V or SB. **b**, **c** Effects of SB on the average tumor growth of all mice (V; 5 mice and SB; 6 mice). Experiment was repeated three times. **d**, **e** Average tumor volume of MiaPaCa2 and A549 tumors transfected with NT siRNA, KRas siRNA, or GSK3α/β siRNA (NT; 3 mice, KRas sRNA; 3 mice, and GSK3 siRNA; 3 mice). Experiment done once. For **b** and **c**, day 0 corresponds to day 14 after tumor implantation when the average tumor volumes were 150–200 mm^3^; for **d** and **e**, day 0 corresponds to the day when the tumors were implanted. Insets: western blots confirming depletion of KRas and GSK3. **f**–**h** Effects of vehicle and SB on the growth of patient-derived xenografts (PDXs) of chemotherapy/radiation-resistant primary (**f**), chemo-naive primary (**g**), and chemo/radiation-resistant metastatic (**h**) tumors from pancreatic cancer patients. For **f** and **h**, V; 10 mice and SB; 10 mice. For **g**, V; 10 mice and SB; 9 mice) (*P < 0.05; **P < 0.01; ***P < 0.001). *P* values determined by Student’s *t*-test

To identify kinase inhibitors that selectively suppress the viability of human cancer cells that depend on mutant KRas, we first screened a 304-compound, well-cured kinome inhibitor library, the GlaxoSmithKline Published Kinase Inhibitor Set 1^12^, against human pancreatic (MiaPaCa2) and lung (A549) cancer cells. Although both MiaPaCa2 and A549 cell lines harbor mutant KRas, previous work showed these cell lines to be mutant KRas-dependent and -independent, respectively^13,14^, and we confirmed their dependency status in cell culture (Fig. 2) and in mice (Fig. 3). After cells were treated with the 304 kinase inhibitors (1 µM) for 72 h in 96-well plates using a “one well-one kinase inhibitor” format, we determined the difference in percent inhibition of viability [*D* = (% MiaPaCa2 inhibition) − (% A549 inhibition)] for each compound based on the average of two screens. Figure 1a shows that 197/304 compounds (65%) affected MiaPaCa2 and A549 cell viability equally, with *D* values from −10% to +10%. The GSK3α/β inhibitor SB-732881-H (SB) (Fig. 1a, inset) had the highest selectivity for inhibiting the viability of MiaPaCa2 versus A549 cells (*D* = 81%), inhibiting MiaPaCa2 cells by 85 and 80% (two independent screens) but A549 cells by only 1 and 3% (Fig. 1b). We synthesized SB in-house to confirm these findings, showing that SB inhibited MiaPaCa2 versus A549 cell viability with a 45-fold lower IC_50_ (0.4 versus 18 µM; Fig. 1c). Other GSK3 inhibitors (Tideglusib, AZD1080, and BIO) with chemical structures different from SB showed similar selectivity (Supplementary Fig. 1, top), confirming that MiaPaCa2 cells are more sensitive than A549 cells to inhibition of GSK3α/β. Furthermore, SB induced caspase-3 activation and PARP cleavage only in MiaPaCa2 but not in A549 cells (Fig. 1d), suggesting that GSK3α/β inhibition selectively induces apoptosis in mutant KRas-dependent tumor cells. Kinome profiling at 100 nM showed that SB is highly selective for GSK3α/β, with over 96% (216/224) of the kinases inhibited by <10% (data from ref. ^9^ plotted as Supplementary Fig. 1, bottom; with permission from *Nature Biotechnology*). GSK3α and GSK3β were the two most potently inhibited kinases (80% and 77%, respectively). Furthermore, unlike GSK3 inhibition with SB that suppresses the viability of the mutant KRas-dependent MiaPaCa2 cells selectively over that of the mutant KRas-independent A549 cells, inhibition of other kinases does not. For example, treating MiaPaCa2 and A549 cells with the PLK1 kinase inhibitor, GSK317314A^12^, or the dual PLK1 and LOK inhibitor, GSK237701A^12^, inhibited equally the viability of both cell lines (Supplementary Fig. 2). The MAPK3 inhibitor, GW301789X^12^, and the ErbB4 kinase inhibitor, GR269666A^12^, had little effects on the viability of neither MiaPaCa2 nor A549 cells (Supplementary Fig. 2). Furthermore, the multi-kinase inhibitor GW780056X (ARK5, KIT, CDK4, HIPK1, CLK2, DYRK1, and CDK2)^12^ inhibited equally the viability of MiaPaCa2 and A549 cells, whereas another multi-kinase inhibitor, GSK619487A (PKC, AKT1, IKK, PKA, AKT2, and AKT3)^12^, had little effect on the viability of either cell line (Supplementary Fig. 2).

These findings support the hypothesis that mutant KRas-dependent human cancer cells are vulnerable to GSK3α/β inhibition and that mutant KRas-dependent cells may require GSK3α/β for tumor survival. To further investigate this hypothesis, we used SMARTpool siGENOME small interfering RNAs (siRNAs) to deplete KRas and GSK3α/β from a panel of eight mutant KRas-harboring human cancer cell lines and then determined the effects of silencing these genes on apoptosis and viability. KRas depletion induced caspase-3 activation and PARP cleavage in MiaPaCa2 and L3.6pl (pancreatic), SW620 (colon), and Calu-6 (lung) cells but did not in A549 and H460 (lung) and DLD-1 and HCT-8 (colon) human cancer cells (Fig. 2a), indicating that the former four tumor cell lines are mutant KRas-dependent, whereas the latter four cell lines are not. Importantly, GSK3α/β depletion only induced apoptosis in cell lines that were mutant KRas-dependent (Fig. 2a). Similarly, GSK3α/β depletion only inhibited viability in cell lines where depletion of KRas inhibited viability (Supplementary Fig. 3a). Consistent with these findings, pharmacological inhibition of GSK3α/β with SB only induced apoptosis and inhibited viability in cell lines where depletion of KRas induced apoptosis and inhibited viability (Fig. 2b and Supplementary Fig. 3b). Notably, SB did not affect the viability of human non-malignant immortalized cells from kidney (HEK293), pancreas (human pancreatic epithelial nestin-expressing; HPNE), and ovarian (T80) origin (Supplementary Fig. 3b). In addition, SB was not able to induce apoptosis in eight human cancer cell lines that harbor wild-type KRas (H661, H2126, H322, H1299, H522, PC9, H4006, and DU145) as compared to those that harbor mutant KRas (Supplementary Fig. 3c). Finally, Supplementary Fig. 3d shows that SB treatment induced apoptosis in control empty vector cells as well as cells ectopically expressing wild-type GSK3. In contrast, ectopic expression of the constitutive active GSK3-S9A mutant compromised the ability of SB to induce apoptosis. Together, the above findings support the hypothesis that mutant KRas-dependent human cancer cells are vulnerable to GSK3α/β inhibition and that, in these cells, mutant KRas requires GSK3α/β to maintain tumor cell survival.

### GSK3 or KRas silencing inhibits in vivo growth of KRas-dependent tumors

To evaluate the effects of SB on tumor growth in vivo, we subcutaneously implanted MiaPaCa2 and A549 cells on right and left flanks, respectively, of nude mice (Fig. 3a). After tumors reached 150–200 mm^3^, mice were treated daily with vehicle or SB (50 mg/kg intraperitoneally). SB treatment suppressed the growth of MiaPaCa2 but not A549 tumors (Fig. 3a–c). At day 21, MiaPaCa2 tumor growth was inhibited by 81% in SB-treated mice compared with vehicle-treated mice (Fig. 3b), whereas growth of A549 tumors was not inhibited (Fig. 3c). The difference in the average tumor volume between vehicle- and SB-treated MiaPaCa2 tumors was statistically significant starting at day 7 and remained so thereafter (Fig. 3b).

To determine whether depletion of either GSK3α/β or KRas affects MiaPaCa2 and A549 tumor growth in mice, we transfected MiaPaCa2 and A549 cells with non-targeting (NT), KRas, or GSK3α/β siRNA for 48 h and then subcutaneously implanted equal numbers of harvested cells on right (MiaPaCa2) and left (A549) flanks. An aliquot from the same cells used for implantation was analyzed by western blot to confirm depletion (Fig. 3d, e, insets). Compared with control NT siRNA, knockdown of KRas in MiaPaCa2, but not in A549, cells inhibited tumor growth in mice, confirming in vivo the dependency of MiaPaCa2 but not A549 tumors on KRas. Importantly, depletion of GSK3α/β inhibited MiaPaCa2 but not A549 tumor growth, further supporting the hypothesis that mutant KRas-dependent but not -independent tumors require GSK3α/β for growth and survival. Differences in average tumor volume of NT versus KRas or GSK3α/β-depleted MiaPaCa2 tumors were statistically significant starting at day 4 (Fig. 3d, e).

### SB inhibits growth of mutant KRas xenografts from pancreatic cancer patients

To determine whether the GSK3 inhibitor can also inhibit growth of patient-derived xenografts (PDXs), we used fresh tumor biopsies from three pancreatic cancer patients. Patient 1 (G12C mutant KRas; poorly differentiated T3N1 stage IIB) was refractory to neoadjuvant FOLFIRINOX (oxaliplatin (Eloxatin^®^), leucovirin, irinotecan, and fluorouracil)/radiation; patient 2 (G12V mutant KRas; poorly differentiated T3N1 stage IIB) had no therapy; and patient 3 (G12D mutant KRas; stage IV hepatic metastasis) progressed after gemcitabine (Gemzar)/abraxane (albumin-bound or nab-paclitaxel)/xeloda (Capecitabine) with radiation. Freshly resected tumors were subcutaneously implanted in NSG mice as previously described^15^ and randomized into vehicle (*n* = 10) or SB (*n* = 9–10) groups for each of the PDXs. Throughout treatment, SB significantly inhibited tumor growth of all three PDXs (Fig. 3f–h). By the last day of vehicle treatment, PDXs from patients 1, 2, and 3 showed average growth of 531%, 492%, and 522%, respectively. In contrast, SB treatment resulted in only 179%, 232%, and 207% growth, respectively (Fig. 3f–h). Differences in tumor growth between vehicle- and SB-treated mice were statistically significant starting at day 3 (patient 1) and day 2 (patients 2 and 3) (Fig. 3f–h and Supplementary Fig. 4a–c).

### SB treatment of mice blocks c-Myc and β-catenin phosphorylation

**Fig. 4:**
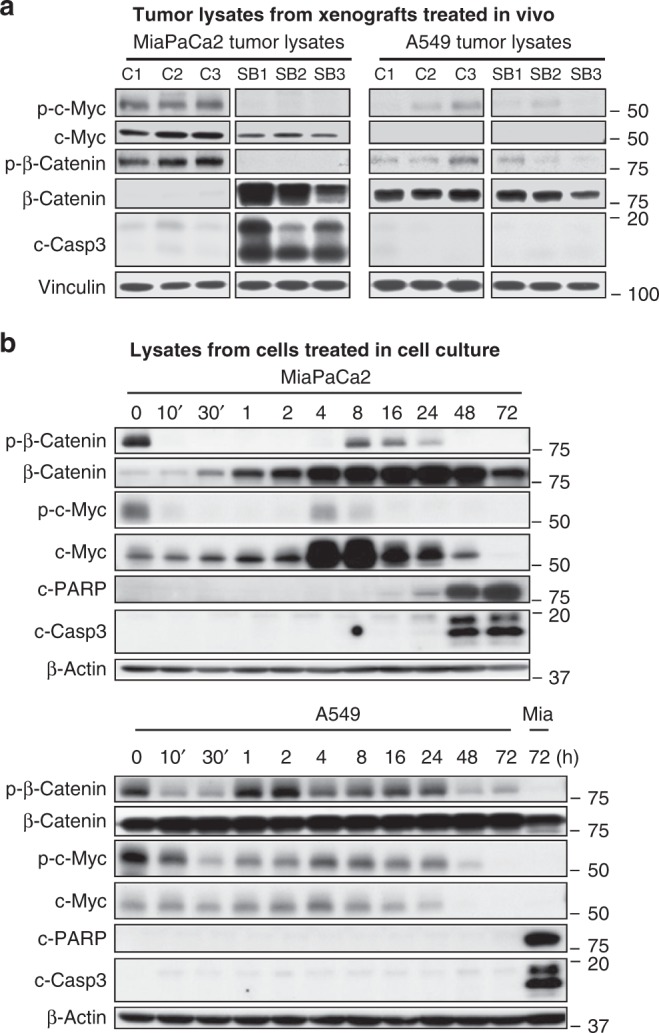
SB blocks the phosphorylation of β-catenin S33/S37/T41 and T58 c-Myc. **a** Western blots of MiaPaCa2 and A549 tumor lysates from xenografts resected from mice treated with vehicle or SB. C1, C2, and C3 are tumors from mice treated with vehicle control. SB1, SB2, and SB3 are tumors from mice treated with SB. Experiment was done once. **b** Western blot of MiaPaCa2 and A549 cells treated with 1 µM SB for various lengths of time. Mia designates MiaPaCa2 lysates used as positive control. Experiment was done twice

To determine whether SB inhibited its target in vivo, we evaluated whether SB affected phosphorylation of the GSK3 substrates c-Myc and β-catenin in MiaPaCa2 and A549 xenografts. As shown in western blots, MiaPaCa2 but not A549 tumors from vehicle-treated mice contained high levels of phosphorylated c-Myc (Fig. 4a), as measured by an antibody that recognizes phospho-T58 c-Myc. T58 on c-Myc is phosphorylated by GSK3^16^. Phosphorylation of c-Myc on T58 was blocked in MiaPaCa2 tumors from mice treated with SB, indicating that SB reached its target in vivo. Similar to the c-Myc results, MiaPaCa2, but not A549, tumors from vehicle-treated mice contained high levels of phosphorylated β-catenin, as measured by an antibody that recognizes phospho-S33/S37/T41 β-catenin (Fig. 4a). β-Catenin phosphorylation by GSK3 on S33, S37, and T41 primes it for ubiquitination by the βTrCP E3 ubiquitin ligase and destruction by the proteasome^17^. Accordingly, we observed total β-catenin levels to be extremely low in MiaPaCa2 but not in A549 tumors (Fig. 4a). Phosphorylation of β-catenin on S33, S37, and T41 was blocked in MiaPaCa2 tumors from mice treated with SB, resulting in increased accumulation of β-catenin. Importantly, and consistent with our cell culture results, SB treatment induced apoptosis in MiaPaCa2 but not in A549, tumors in vivo (Fig. 4a).

### c-Myc and β-catenin contribute to antitumor activity of GSK3 blockade

The correlation between tumor growth inhibition, induction of apoptosis, and inhibition of c-Myc and β-catenin phosphorylation in MiaPaCa2 tumors in vivo suggested that c-Myc and/or β-catenin may contribute to the antitumor activity of SB. To test this hypothesis, we determined the effects of SB treatment (1 µM) in MiaPaCa2 and A549 cells on phospho-β-catenin, phospho-c-Myc, and their corresponding total levels. Consistent with in vivo results (Fig. 4a), MiaPaCa2 cells had high levels of phospho-S33/S37/T41 β-catenin and little total β-catenin (Fig. 4b). In addition, β-catenin phosphorylation was rapidly (within 10 min) blocked by SB, and this blockade was paralleled by an accumulation of total β-catenin levels. A small and transient increase in phospho-β-catenin occurred between 8 and 24 h, but the increase in total β-catenin was maintained. Similarly, SB rapidly decreased phospho-T58-c-Myc levels, which was paralleled by a modest increase in total c-Myc in the first 2 h followed by substantial increases at 4 and 8 h. This is consistent with the observation that phosphorylation of T58 by GSK3 is associated with c-Myc degradation^16^. In contrast to the increase in β-catenin levels that was maintained until 72 h, total c-Myc decreased after 8 h and was barely detectable by 72 h (Fig. 4b). This is consistent with in vivo results where long-term (21 days) SB treatment led to lower levels of c-Myc (Fig. 4a). In A549 cells, SB transiently decreased phospho-β-catenin at 10 and 30 min but this had little effect on the already high levels of total β-catenin (Fig. 4b). SB decreased phospho-c-Myc levels but had little effect on total levels, except a barely detectable effect after 24 h. Consistent with the results shown in Figs. 1d, 2b, and 4a, SB induced apoptosis in MiaPaCa2 but not in A549 cells (Fig. 4b).

**Fig. 5:**
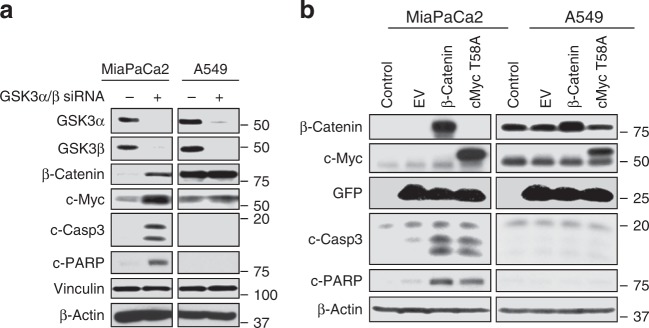
**a** GSK3α/β depletion induces β-catenin and c-Myc in MiaPaCa2 but not in A549 cells. The cells were transfected with GSK3-α/β siRNA or NT siRNA for 72 h and processed for western blotting with the indicated antibodies. Experiment was done three times. **b** Ectopic expression of CA β-catenin or T58A c-Myc induces apoptosis in MiaPaCa2 but not in A549 cells. Cells were infected with lentiviruses that contain mutant β-catenin that lacks the GSK3 phosphorylation sites, S33/S37/T41, and is therefore constitutively active, or mutant T58A c-Myc that cannot be phosphorylated by GSK3; cells were processed for western blotting after 96 h. Experiment was done three times

To further confirm whether the effects of SB on c-Myc and β-catenin levels are due to inhibition of GSK3, we have shown that depletion of GSK3α/β with siRNA phenocopies the pharmacological inhibition of GSK3α/β with SB by upregulating c-Myc and β-catenin in MiaPaCa2 but not in A549 cells (Fig. 5a). We further reasoned that if SB inhibition of β-catenin and c-Myc phosphorylation contributes to apoptosis induction, then forced expression of phosphorylation-deficient mutants of β-catenin and c-Myc should induce apoptosis in MiaPaCa2 but not in A549 cells. To this end, we infected these cells with GFP-lentivirus constructs containing mutant β-catenin that lacks the S33, S37, and T41 GSK3 phosphorylation sites and is therefore constitutively active^18^ or a T58A c-Myc mutant that cannot be phosphorylated by GSK3. After 96 h, western blots showed that ectopic expression of mutant β-catenin and mutant c-Myc induced apoptosis in MiaPaCa2 but not in A549 cells (Fig. 5b).

**Fig. 6:**
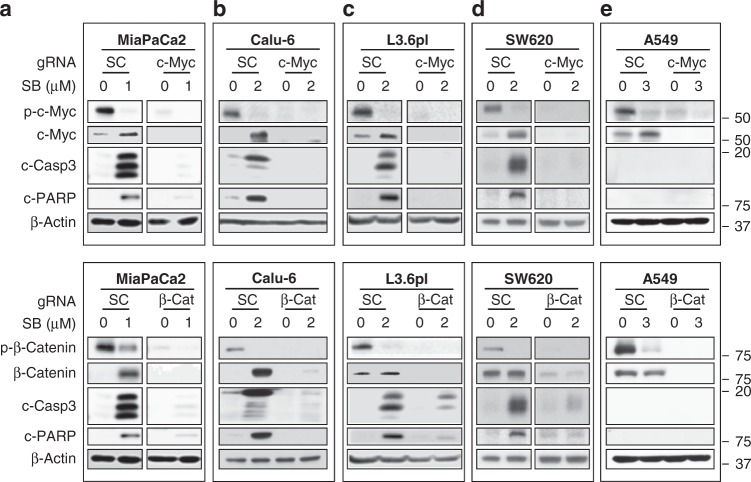
CRISPR/Cas9 knockout of β-catenin or c-Myc rescues from SB-induced apoptosis. **a**–**e** Western blots of MiaPaCa2 (**a**), Calu-6 (**b**), L3.6pl (**c**), SW620 (**d**), and A549 (**e**) cells treated with SB at indicated concentrations for 48 h after knocking out c-Myc (top panel) or β-catenin (bottom panel) with lentiviral guide RNAs (gRNAs), including scrambled (SC), β-catenin (β-Cat), and c-Myc gRNAs. Experiments were repeated at least three times for all cell lines except for SW620 and A549 where they were done once

To investigate whether SB-induced apoptosis is mediated by c-Myc and/or β-catenin, we determined whether CRISPR/Cas9-targeted knockout of c-Myc or β-catenin rescues MiaPaCa2 cells from SB-induced apoptosis. After MiaPaCa2 cells were infected with lentivirus containing Cas9 and guide RNA (gRNA) to either β-catenin or c-Myc or scrambled for 48 h and subjected to antibiotic selection, we treated the resulting stable cells with SB for 48 h. In scrambled gRNA-infected cells, SB inhibited c-Myc and β-catenin phosphorylation, upregulated c-Myc and β-catenin, and induced caspase-3 activation and PARP cleavage (Fig. 6a). In contrast, targeted knockout of c-Myc and β-catenin in cells infected with c-Myc or β-catenin gRNA abrogated SB-induced apoptosis (Fig. 6a). Similar results were obtained with the other mutant KRas-dependent cells (Calu-6, L3.6pl, and SW620) where knocking out c-Myc or β-catenin prevented SB from inducing apoptosis (Fig. 6b–d). Unlike mutant KRas-dependent cells, in A549 cells, SB did not induce apoptosis in scrambled gRNA-infected cells or in c-Myc- and β-catenin-knocked out cells (Fig. 6). Taken together, we found that pharmacological inhibition and gene silencing of GSK3α/β selectively induced apoptosis and inhibited tumor growth of mutant KRas-dependent tumors, which was at least in part mediated by c-Myc and β-catenin.

## Discussion

In this manuscript, we revealed that human tumors dependent on mutant KRas require GSK3α/β for viability, survival, and tumor growth. In contrast, GSK3α/β is dispensable for mutant KRas-independent tumors. Importantly, the in vivo growth of three PDXs from primary chemo-naive, primary refractory, and metastatic refractory pancreatic cancer patients was inhibited by the GSK3α/β inhibitor SB. These results suggest that SB can overcome resistance to chemo- and radiation therapy since two of the fresh biopsies were resected from patients who progressed on FOLFIRINOX, gemcitabine, abraxane, xeloda, and/or radiation. The antitumor effects of SB were independent of the mutant KRas isoforms, which included G12D, G12V, and G12C (fresh biopsies) and G12D, G12V, G12C, and Q61K (cell lines). This is important as it has been suggested that the position and type of mutations in KRas influence the transforming capacity of mutant KRas proteins and drug responses of cancer patients. It is important to note that GSK3 inhibitors did not score in previous chemical screens. One possible reason for this is that unlike our chemical screen, the previous chemical screens were not designed specifically to identify compounds that inhibit mutant  KRas-harboring/KRas-dependent, but not mt KRas-harboring/KRas-independent human cancer cell lines. The second possible reason is that in all our genetic depletion and pharmacological inhibition studies, we have targeted simultaneously both GSK3α and GSK3β. Indeed, mutant KRas-dependent human cancer cells undergo apoptosis only when both GSK3α and GSK3β are knocked down simultaneously (Supplementary Fig. 5). In contrast, knocking down GSK3α or GSK3β individually did not induce apoptosis (Supplementary Fig. 5).

An important finding of our studies is that suppression of GSK3α/β inhibits mutant KRas-dependent tumor growth at least in part by a c-Myc- and β-catenin-dependent mechanism. Consistent with this, depletion of GSK3α/β increased c-Myc- and β-catenin levels; and forced expression of β-catenin or c-Myc mutants that lack GSK3 phosphorylation sites is sufficient to induce apoptosis. Strikingly, these findings suggest that in mutant KRas-dependent human tumors, GSK3 has pro-survival activity, whereas β-catenin and c-Myc have pro-apoptotic activity. Although the roles for GSK3α/β in promoting apoptosis are well documented, there is also evidence for its pro-survival function. The reasons for this bi-functional role of GSK3 in survival and apoptosis have been thus far poorly understood and mainly explained by context-specific effects regarding cell lineage, signaling circuits, and transformation status^19^. For example, GSK3α/β induces apoptosis during DNA damage, hypoxia, and endoplasmic reticulum stress^19^. However, in some cancers GSK3 is overexpressed^20^, and promotes survival by several mechanisms, including activation of the canonical^21^ and non-canonical^22^ nuclear factor-kB pathways, inhibition of apoptotic signaling and caspase activation^23^, and inhibition of c-Myc-induced apoptosis^24^. Our findings are consistent with a pro-survival role for GSK3 in mutant KRas-dependent tumors, where GSK3 inhibition leads to c-Myc- and β-catenin-dependent tumor suppression.

Although c-Myc overexpression is associated with tumorigenesis, c-Myc can also induce apoptosis^24,25^. For example, c-Myc can induce apoptosis by stabilizing p53 through Foxo/ARF blockade of Mdm2^26^ and by activating ATM kinase^27^, TRAIL^28^, or the pro-apoptotic protein Bax^29^. When we inhibited GSK3 with SB treatment, c-Myc levels were substantially increased within 4 h of treatment, and this coupled with the gene knockout results of Fig. 6, suggest that c-Myc was necessary for SB to induce apoptosis in mutant KRas-dependent human cancer cells.

c-Myc is a known transcriptional target of β-catenin; therefore, SB could induce apoptosis by inhibiting the phosphorylation of β-catenin, leading to its stabilization and induction of c-Myc transcription. This is consistent with the data in Fig. 4b, where the increase in β-catenin is accompanied by modestly increased c-Myc levels at early time points. Alternatively, SB could directly induce c-Myc in a β-catenin-independent manner by inhibiting its GSK3-dependent phosphorylation on T58. The significant increase in total c-Myc levels at 4 and 8 h, beyond the initial modest increase at earlier time points (Fig. 4b), coupled with the fact that T58 phosphorylation promotes c-Myc degradation^16^, support this direct mechanism. Further support for this is provided by the demonstration that the T58A c-Myc mutant is able to induce apoptosis on its own.

Together, these findings suggest that in mutant KRas-dependent human cancer cells, inactivating GSK3α/β induces apoptosis in a β-catenin- and c-Myc-dependent manner. Our findings also suggest that during progression of some tumors, mutant KRas may lead to a GSK3α/β-dependent downregulation of β-catenin and/or c-Myc and that inhibition of GSK3 in these cells unleashes the pro-apoptotic action of β-catenin and/or c-Myc. In contrast, GSK3α/β inhibition did not upregulate β-catenin and c-Myc and did not induce apoptosis in mutant KRas-independent human tumors.

In summary, the novel mechanism presented here opens new avenues to therapeutically target mutant KRas-dependent human cancers by suppressing GSK3α/β, leading to β-catenin- and/or c-Myc-dependent tumor abrogation. This significant finding has the potential to overcome the challenges that mutant KRas poses as a prevalent driver of human oncogenesis and therapy resistance. The GSK3α/β inhibitor sensitivity of xenografts from primary and metastatic pancreatic cancer patients who progressed on chemo- and radiation therapies warrants further clinical investigation of GSK3α/β inhibitors as single agents or in combination.

## Methods

### Cells lines, cell culture, and reagents

Human lung (Calu-6, A549, H460, H661, H2126, H322, H1299, H522, PC9, and H4006), colon (SW620, DLD-1, and HCT-8), pancreatic (MiaPaCa2 and L3.6pl), and prostate (DU145) cancer cell lines and HEK293 cells were obtained from the American Type Culture Collection and cultured in Dulbecco’s modified Eagle’s medium (DMEM) or RPMI-1640 medium. Normal/immortalized T80 cells (J. Liu and R. Bast, MD Anderson Cancer Center) were cultured in Medium 199/MCDB 105. hTERT-immortalized HPNE cells (Channing Der, University of North Carolina) were grown in medium D (mixture of M3 medium and DMEM) containing one volume of M3 Base F culture medium (InCell Corp.), three volumes of glucose-free DMEM, 5.5 mM glucose, 10 ng/mL EGF, and 50 µg/mL gentamycin. All media were supplemented with 10% heat-inactivated fetal bovine serum, 10 U/mL penicillin, and 10 µg/mL streptomycin. SB was synthesized in-house as described previously^30^ and dissolved in dimethyl sulfoxide (DMSO; Sigma-Aldrich). Tideglusib, AZD1080, and BIO were purchased from SelleckChem. The Published Kinase Inhibitor Set 1 (PKIS1) of 304 compounds was received from GlaxoSmithKline (GSK). All cell lines were mycoplasma-free, monitored regularly with HEK-blue2 cells and mycoplasma detection kit from invivogen (cat# rep-pt1).

### Screening of PKIS1 library

KRas-dependent (MiaPaCa2) and -independent (A549) human cancer cell lines were screened with the 304 kinase inhibitor compounds of PKIS1 library to identify a kinase inhibitor that can selectively inhibit the viability of MiaPaCa2 over A549 cells using MTT assays. Cells were cultured in 96-well plates at a density of 3 × 10^3^ cells/well and allowed to adhere overnight. The medium was then replaced with medium containing vehicle (0.2% DMSO) or 1 µM of each of 304 compounds for 72 h in one compound-one well format. After cells were incubated with 1 mg/mL MTT (Sigma), cell viability was quantified as described previously^31^.

### Western blot analysis

To prepare whole-cell lysates, cells were washed twice with phosphate-buffered saline (PBS) and lysed on plates in Mammalian Protein Extraction Reagent (Thermos Scientific, catalog no. 78501) supplemented with protease inhibitor cocktail, 2 mM phenylmethylsulfonyl fluoride, 2 mM Na_3_VO_4_, and 6.4 mg/mL *p*-nitrophenylphosphate. Proteins from the lysates were separated by SDS-polyacrylamide gel electrophoresis and western blotted with the following antibodies: (a) from Santa Cruz: β-catenin (H-102; catalog no. sc-7199) and c-Myc (9E10, sc-40); (b) from Abcam: phospho-c-Myc (phospho-T58) (ab185655); (c) from Cell Signaling: GSK3α (D80E6; catalog no. 4337), GSK3β (27c10; catalog no. 9315), cleaved caspase-3 (catalog no. 9664L), cleaved PARP (catalog no. 5625S), phospho-β-catenin (Ser 33/37/Thr41; catalog no. 9561); (d) from Calbiochem: anti-c-KRas (Ab-1; catalog no. OP24); and (e) from Sigma-Aldrich: vinculin (catalog no. V9131-.2ML) and anti- β-actin (catalog no. A5441).

### Vector constructions and lentivirus production

Mutant T58A c-Myc was generated by mutagenesis PCR using forward primer T58A-F (TGCTGCCCGCCCCGCCCCTGTCCCCTAGCCGCC) and reverse primer T58A-R (GGGGCGGGGCGGGCAGCAGCTCGAATTTC) (see Table 1 in the Supplementary Information) and wild-type c-Myc from MiaPaCa2 cells as template. For overexpression studies, T58A c-Myc was inserted in lentiviral pLEX-3FLAG-T2A-GFP vector (modified from pLEX-MCS vector from Openbiosystem) between *Not*I and *Bam*HI sites. Mutant β-catenin (pcDNA3 deltaN47 β-catenin, Addgene plasmid #19287) was amplified by PCR and also constructed in pLEX-3FLAG-T2A-GFP vector between *Not*I and *Bam*HI sites.

To knockout c-Myc and β-catenin in human cancer cell lines, we constructed gRNAs in LentiCRISPRv2 vector (addgene #52961 and #83480) as described previously^32^ and tested their knockout efficiency in MiaPaCa2 cells by western blot. The most efficient gRNA vector was selected for knocking out c-Myc (gRNA sequence: CTGCTCGCCCTCCTACGTTG) and β-catenin (gRNA sequence: TCCCACTAATGTCCAGCGTT) in all cell lines (see Table 1 in the Supplementary Information). The scramble gRNA (GCACTACCAGAGCTAACTCA) was also inserted into LentiCRISPRv2 vector and served as negative control for all cell lines.

For lentivirus production, the lentiviral vector (10 µg) and the two packaging viral vectors, pMD.2G (5 µg) and pspPax2 (5 µg), were co-transfected into 293T cells using polyethylenimine (25 kDa, 1 µg/µL)^33,34^. The virus supernatant was collected at 48 h and concentrated at 1:100 ratio using Lenti-X™ Concentrator (TaKaRa/Clontech, #631231). Viral RNA genome copies/mL were determined using Lenti-X™ qRT-PCR Titration Kit (TaKaRa/Clontech).

### gRNA-mediated knockout of β-catenin and c-Myc and SB rescue experiments

To generate stable pools, the cells infected with lentivirus containing scrambled gRNA, c-Myc gRNA, or β-catenin gRNA were selected with antibiotics (puromycin or blasticidin) for 5 days before pooling together and SB treatments. To determine whether knocking out c-Myc or β-catenin rescues from SB-induced apoptosis, these stable pools were seeded into six-well plates at 0.3 × 10^6^ cells/well and treated with SB for 48 h at 1 μM for MiaPaCa2, 2 μM for Calu-6, L3.6pl, and SW620, and 3 μM for A549 cells. It is important to use early passage (passage 1 or 2 after stable pool generation) for these experiments. Western blots were performed as described above to detect phospho-β-catenin (Ser 33/37/Thr41), β-catenin, phospho-c-Myc (T58), c-Myc, cleaved PARP, cleaved caspase-3, and β-actin.

### siRNA-mediated knockdown of KRas and GSK3α/β

After cells were plated onto 6-well plates for western blotting and then 96-well plates for cell viability assays for 24 h, they were transiently transfected using the Lipofectamine RNAiMAX reagent (catalog no. 13778; Invitrogen, Carlsbad, CA) with 20 nM SMARTpool Human KRas siRNA (catalog no. M-005069-00; Dharmacon), SMARTpool NT siRNA #2 (catalog no. D-001206-14-05; Dharmacon), SignalSilence^®^ GSK3α/β siRNA (catalog no. 6301; Cell Signaling), and SignalSilence^®^ control siRNA (unconjugated; catalog no. 6568; Cell Signaling). Transfected cells were collected 48 or 72 h after transfection for western blot analysis as described above, and after 72 h for CellTiter-Glo^®^ Luminescent Cell Viability Assay (Promega, Madison, WI, USA) as described below.

### Cell viability assay

Cell viability assays were carried out using the CellTiter-Glo^®^ Luminescent Cell Viability Assay (Promega, Madison, WI). Cells were seeded in 96-well plates at a density of 3 × 10^3^ to 4 × 10^3^ cells/well, allowed to adhere overnight, and treated with vehicle (DMSO) or drug for 72 h, after which they were processed for viability using CellTiter-Glo reagent. Each condition was performed in replicates of 6 wells.

### Antitumor studies of human tumor xenografts in nude mice

Female athymic nude mice were purchased from Charles River Laboratories (Wilmington, MA). The mice were housed, maintained, and treated in accordance with the Institutional Animal Care and Use Committee procedures and guidelines. Exponentially growing A549 and MiaPaCa2 cells were harvested via trypsinization, pelleted at 300 × *g* for 5 min, resuspended in sterile Dulbecco’s PBS (DPBS; Invitrogen) at 5 × 10^6^ cells (MiaPaCa2) and 10 × 10^6^ (A549) cells per 100 µL, and injected into right and left flank, respectively, of each mouse. The tumor xenografts were monitored with an electronic caliper three times per week. Tumor volume was calculated using the following formula: volume = (*L*^2^*W*)/2, where *L* is length and *W* is width, with width defined as the largest measurement and length is the smallest measurement. When the tumors reached 150–200 mm^3^, animals were randomized and treatment schedules (control or SB) were implemented. The vehicle received 10% DMSO, 50% PEG (MW 300 from MP Biochemicals, LLC, Solon, OH), and 40% dH2O; the SB group received 50 mg/kg/day of SB-maleic acid salt. Vehicle and SB (100 µL) were administered by intraperitoneal injections once daily.

To determine the effects of depleting KRas and GSK3α/β on the growth of MiaPaCa2 and A549 cells, these cells were transiently transfected with 20 nM of SMARTpool siGENOME human KRas, GSK3α/β, and NT siRNA as described above for 48 h, then harvested, washed twice in ice-cold sterile DPBS (Invitrogen), counted for viable cells by trypan blue exclusion, resuspended in sterile DPBS at 5 × 10^6^ cells (MiaPaCa2) and 10 × 10^6^ (A549) cells/100 µL, and injected into right and left flanks, respectively, of each mouse. A portion of these transiently transfected cells were collected to run for western blot analysis to confirm the knockdown of target genes by siRNA. The tumor xenografts were monitored, and the volumes were determined three times a week as described above.

### Antitumor efficacy studies of PDXs of tumors from pancreatic cancer patients

To determine whether pharmacological inhibition of GSK3 can inhibit the growth of PDXs, we obtained fresh tumor biopsies from three pancreatic cancer patients (University of Florida, IRB protocol # 201600873). Patient 1 (G64) (G12C KRas mutation), who showed poorly differentiated T3N1 pancreatic cancer (stage IIB), completed 6 weeks of neoadjuvant FOLFIRINOX with 1 week of radiation. Therapy was concluded 5 weeks before surgery. CA 19-9 biomarker levels declined from 500 to 74 during this time, but final pathology demonstrated no tumor necrosis (poor histopathologic response). Patient 2 (G80) (G12V KRas mutation), who also showed poorly differentiated T3N1 pancreatic cancer (stage IIB), received no neoadjuvant therapy. Patient 3 (LM3) (G12D KRas mutation), who had hepatic metastatic pancreatic cancer (stage IV), progressed after neoadjuvant therapy with gemcitabine, abraxane, and xeloda with radiation. Therapy duration was 6 weeks and was completed 6 weeks before surgery.

Upon pancreatic tumor resection, fresh 2-mm tumor pieces were obtained and transported on ice to the animal surgery suite for subcutaneous implantation into NOD.Cg-Prkdcscid Il2rgtm1Wjl/SzJ (NSG) mice. The mice were housed, maintained, and treated in accordance with the Institutional Animal Care and Use Committee procedures and guidelines (protocol # 201406590). Incisions of 1 cm were made on right flanks of anesthetized NSG mice, and blunt dissection of the subcutaneous layer was performed. A viable tumor piece was placed in the flank subcutaneous tissue, and the skin was closed with surgical clips (generation 1). Once engrafted and tumors reached end point (1.5 cm in diameter), tumors were divided evenly into 2-mm pieces and re-implanted into NSG mice as above (generation 2). Generation 3 was generated similarly (details as described in ref. ^15^). When the tumors from early-passage (generation 2 or 3) mice reached approximately 100–200 mm^3^, the animals were randomized into vehicle and SB treatment groups as described above for the cell line xenograft models.

### Ectopic expression of β-catenin and c-Myc

The day before infection, 2 × 10^5^ cells were seeded per well of six-well plates. Cells were plated overnight to reach 70–80% confluence and were then infected with concentrated lentiviral (200 × 10^8^ copies total) GFP-EV, GFP mutant β-catenin^35^, and mutant GFP-T58A c-Myc^25,36^. Infected cells were harvested after 96 h and processed for western blot analysis.

### Statistical tests

All *P* values in this manuscript were determined by Student’s *t*-test. All error bars in figures represent standard errors.

## Electronic supplementary material

Reporting Summary

Supplementary Information

## Data Availability

All data generated or analyzed during this study are included in this published article (and its Supplementary Information files).
