# Supplementary material for: GSK3 suppression upregulates β-catenin and c-Myc to abrogate KRas-dependent tumors
Source: Nat Commun. 2018 Dec 4;9:5154. doi: 10.1038/s41467-018-07644-6 (PMC6279809; doi:10.1038/s41467-018-07644-6)

Supplementary Information

GSK3 Suppression Upregulates β-Catenin and c-Myc to Abrogate KRas-Dependent Tumors

Kazi et al.

Supplementary Figure 1

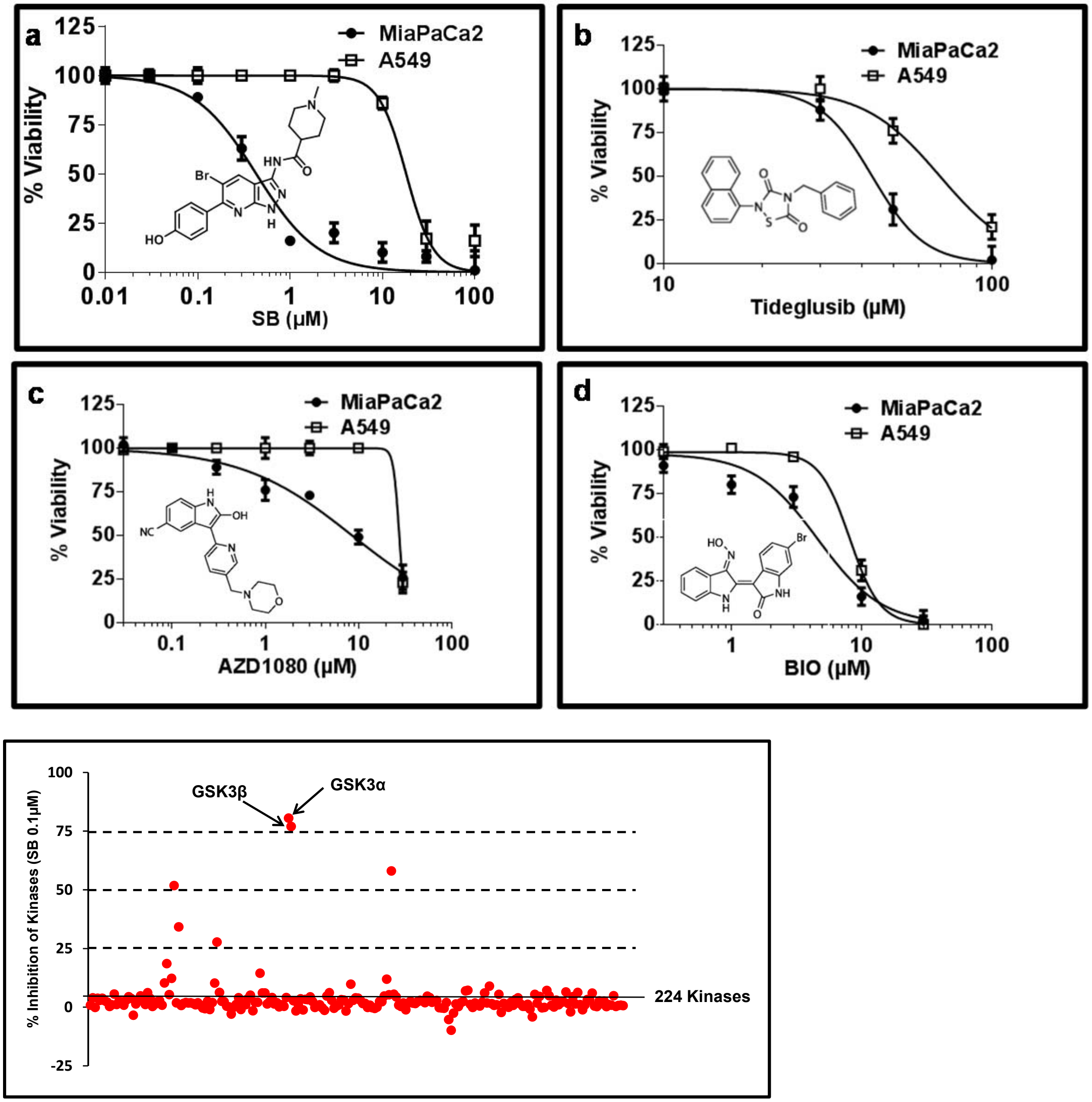

Supplementary Figure 1. GSK3 inhibitors SB (a), Tideglusib (b), AZD1080 (c), and BIO (d) inhibited viability selectively in MiaPaCa2 over A549 cancer cells. MiaPaCa2 and A549 cells were treated for 72 hours with various concentrations of SB, Tideglusib, AZD1080, and BIO and processed for viability assays. Each experiment was done 3 times. Bottom panel shows that the GSK3 inhibitor SB is potent and selective for GSK3α and GSK3β in a panel of 224 kinases.

Supplementary Figure 2.

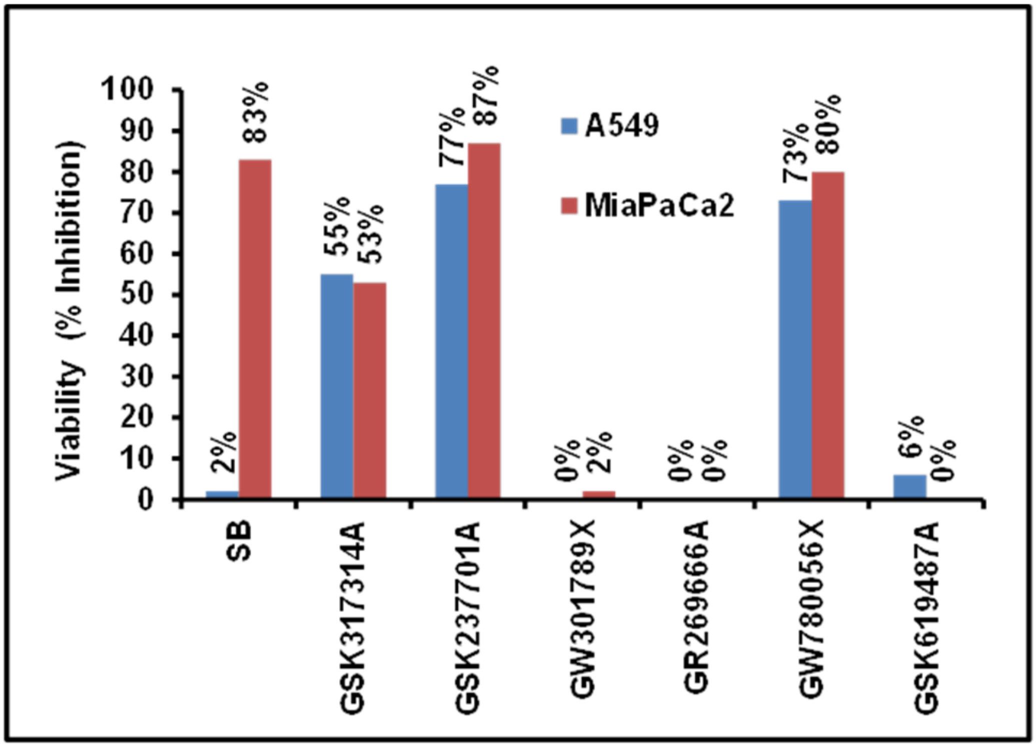

Supplementary Figure 2. SB but not other kinase inhibitors inhibited the viability of MiaPaCa2 selectively over A549 cancer cells. MiaPaCa2 and A549 cells were treated for 72 hours with 1 uM of GSK3 inhibitor SB; PLK1 kinase inhibitor GSK317314A; dual PLK1 and LOK inhibitor, GSK237701A; MAPK3 inhibitor, GW301789X; ErbB4 kinase inhibitor GR269666A; multi-kinase inhibitor GW780056X (ARK5, KIT, CDK4, HIPK1, CLK2, DYRK1, CDK2); and multi-kinase inhibitor, GSK619487A (PKC, AKT1, IKK, PKA, AKT2, AKT3); and processed for viability assays.

Supplementary Figure 3.

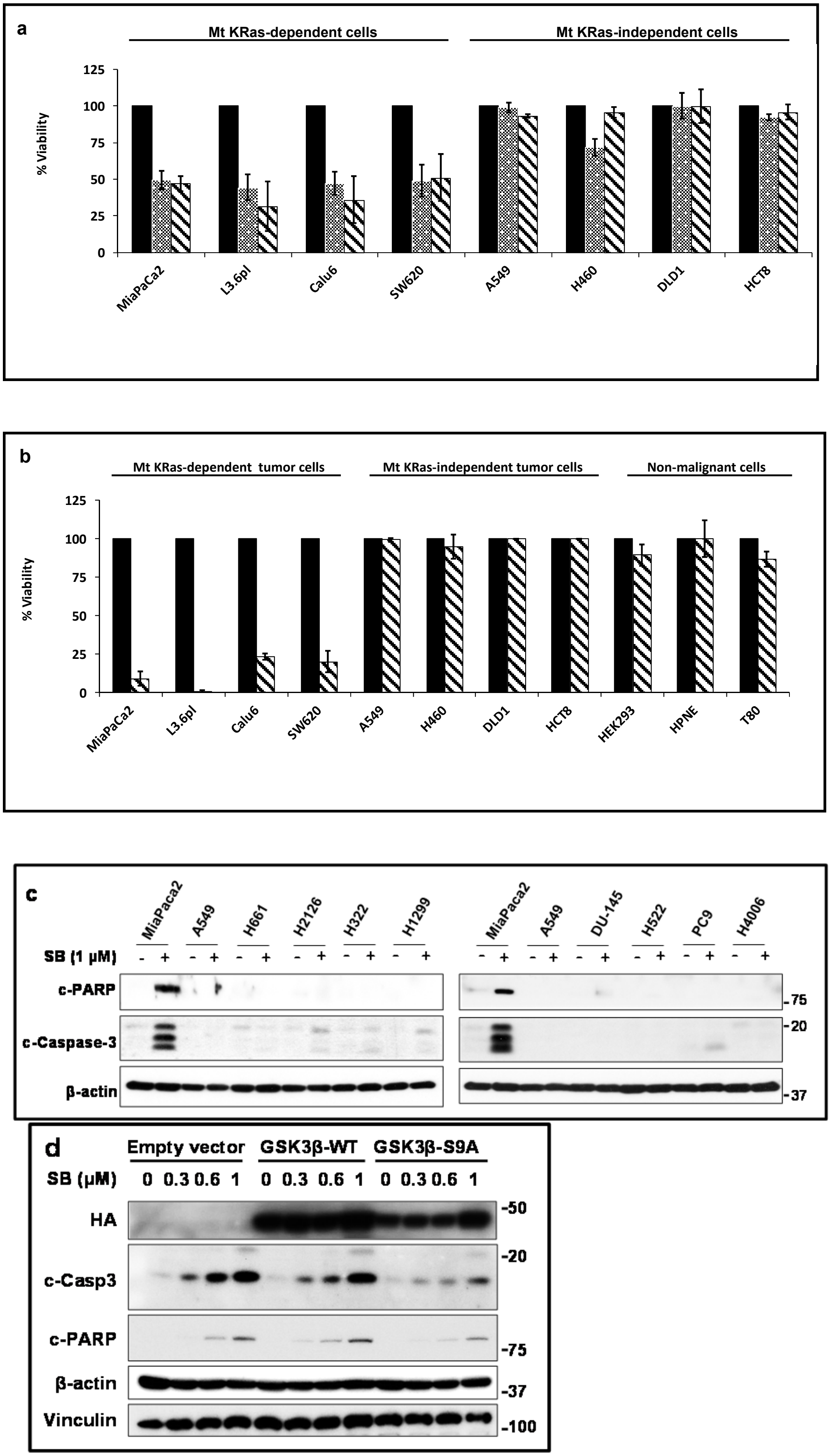

Supplementary Figure 3. Silencing of GSK3α/β or mutant (Mt) KRas selectively compromises the viability of mutant KRas-dependent human cancer cells. (a) Human cancer cell lines were transiently transfected with SMARTpool human KRas siRNA (shaded bars), GSK3-α/β siRNA (hatched bars), or NT siRNA (solid bars) and then processed for viability assays after 72 hours. Experiments were done 3 times for all cell lines except for Calu-6 where it was done twice. **(b)** GSK3α/β inhibitor SB selectively inhibited the viability of mutant KRas-dependent cancer cells but not of mutant KRas-independent cancer cells and "normal" immortalized cells from kidney (HEK 293), pancreas (HPNE), and ovarian (T80) origins. Cells were treated for 72 hours with vehicle (solid bars) or SB (hatched bars) and processed for viability assays. Experiments were done 3 times for all cell lines. (**c**) SB does not induce apoptosis in human cancer cells that harbor wild type KRas. Mutant KRas human cancer cells (MiaPaCa2 and A549) as well as wild type KRas human cancer cells (H661, H2126, H322, H1299, DU-145, H522, PC9 and H4006) were treated with the GSK3α/β inhibitor SB (1 uM) for 48 hours and then processed for Western blotting for c-caspase 3, c-PARP and beta-actin. (**d**) Constitutive active GSK3 S9A mutant compromises the ability of SB to induce apoptosis. MiaPaCa2 cells stably transfected either with empty vector (pcDNA3.1-HA), HA-tagged wild type GSK3 (pcDNA3.1-HA-GSK3-WT) or constitutive active HA-tagged GSK3 S9A mutant (pcDNA3.1-HA-GSK3-S9A) were treated with SB at various concentrations for 48 hours and then processed for western blotting with antibodies against HA, cleaved caspase 3 (c-Casp3), cleaved PARP (c-PARP), b-actin and vinculin.

Supplementary Figure 4.

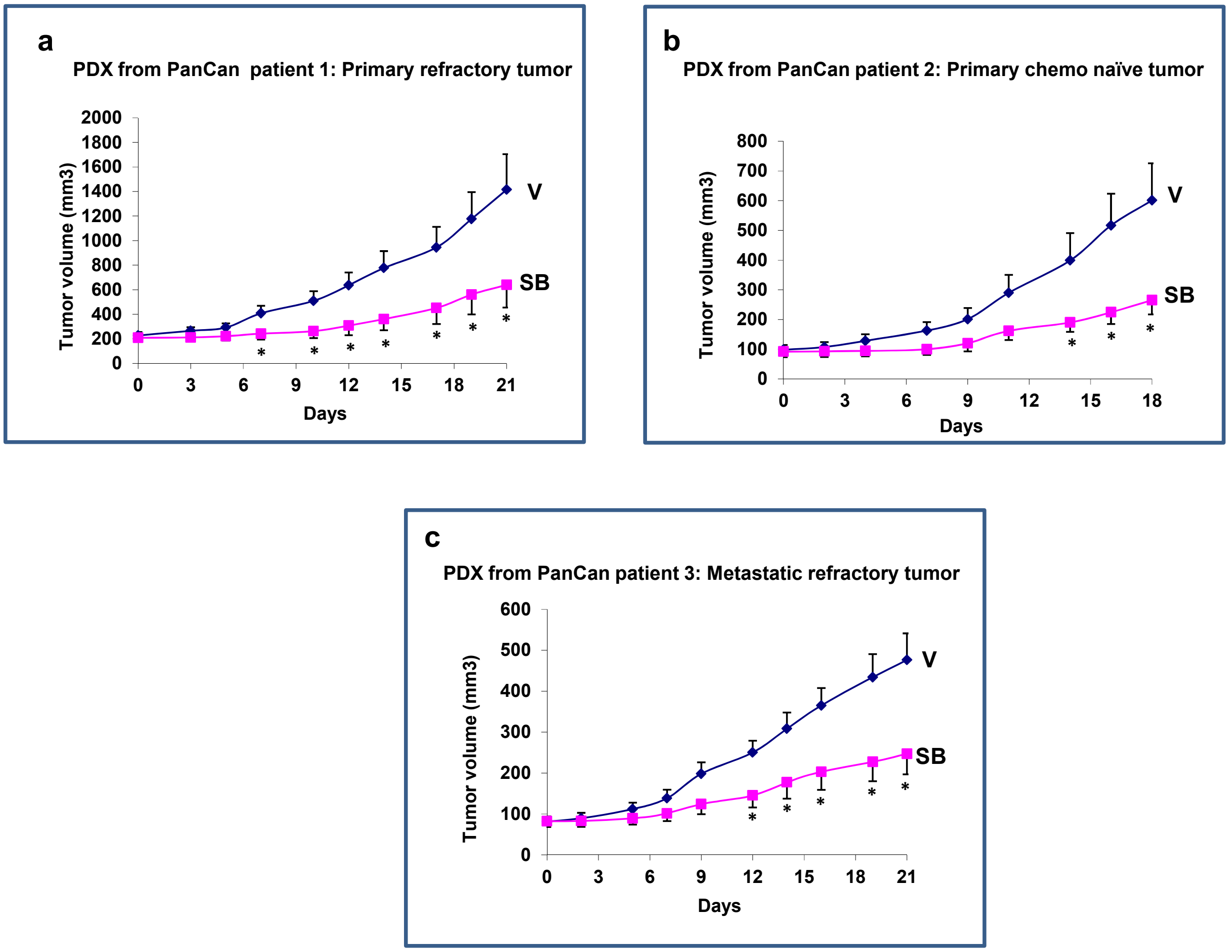

Supplementary Figure 4. GSK3α/β inhibitor SB inhibits PDX tumors from pancreatic cancer patients. Effects of intraperitoneal daily treatments with vehicle (V) or SB (50 mpk) on the growth of patient-derived xenografts (PDX) of primary chemotherapy/radiation therapy-resistant (**a**), primary chemo-naïve (**b**), and chemo/radiation-resistant metastatic (**c**) tumors resected from pancreatic cancer patients. For (**a**) and (**c**); V; 10 mice and SB; 10 mice. For (**b**), V; 10 mice and SB; 9 mice).

Supplementary Figure 5.

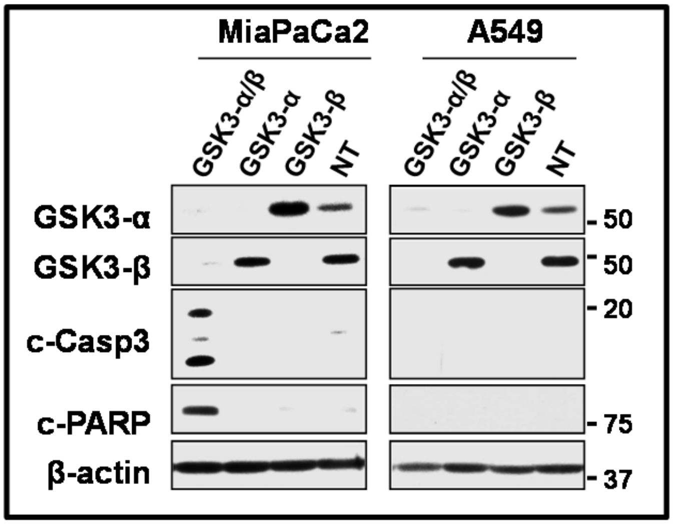

Supplementary Figure 5. Silencing of GSK3α and GSK3β simultaneously, but not individually, induces apoptosis only in mutant (Mt) KRas-dependent human cancer cells. Mutant KRas-dependent (MiaPaCa2) and mutant KRas-independent (A549) human cancer cells were transiently transfected with SMARTpool GSK3α/β, GSK3α, GSK3β or NT siRNAs and processed 72 hours later for Western blotting for GSK3α, GSK3β, c-caspase 3, c-PARP and actin.

Table 1: List of mRNA and guide RNA primer sequences

Primers for Gene expression in pLEX-3FLAG-T2A-GFP vector		
**Gene**	**Forward primer**	**Reverse primer**
β-Catenin	5'-CCCGCGGCCGCGATGGCTACTCAAGCTGATTTG-3'	5'-CCCGGATCCCAGGTCAGTATCAAACCAGG-3'
c-Myc	5'-CCCGCGGCCGCGATGCCCCTCAACGTTAGCTT-3'	5'-CCCGGATCCCGCACAAGAGTTCCGTAG-3'
T58A c-Myc	5'-TGCTGCCCGCCCCGCCCCTGTCCCCTAGCCGCC-3'	5'-GGGGCGGGGCGGGCAGCAGCTCGAATTTC-3'

Primers for Guide RNA expression in Lenti-CRISPRv2 vector		
**guide RNA**	**Forward primer**	**Reverse primer**
sgCatenin	5'-CACCGTCCCACTAATGTCCAGCGTT-3'	5'-AAACAACGCTGGACATTAGTGGGAC-3'
sgMyc	5'-CACCGCTGCTCGCCCTCCTACGTTG-3'	5'-AAACCAACGTAGGAGGGCGAGCAGC-3'
sgSC	5'-CACCGCACTACCAGAGCTAACTCA-3'	5'-AAACTGAGTTAGCTCTGGTAGTGC-3'

Table 1describes the mRNA and guide RNA primer sequences used in this manuscript.

Table 2: List of Antibody dilutions used

Name of antibodies	Catalog Number	Source	Dilution
β -catenin (H-102)	sc-7199	Santa Cruz Biotechnology, Inc	1:50,000
c-Myc (9E10)	sc-40	Santa Cruz Biotechnology, Inc	1:1,000
phospho-c-Myc (phospho T58)	ab185655	Abcam	1:1,000
GSK-3α (D80E6)	4337	Cell Signaling	1:1,000
GSK-3β(27c10)	9315	Cell Signaling	1:1,000
cleaved caspase-3	9664L	Cell Signaling	1:1,000
cleaved PARP	5625S	Cell Signaling	1:1,000
phospho-β-catenin (Ser 33/37/Thr 41)	9561	Cell Signaling	1:1,000
anti-c-KRas (Ab-1)	OP24	Calbiochem	1:1,000
Vinculin	V9131-.2ML	Sigma-Aldrich	1:50,000
anti-β-actin	A5441	Sigma-Aldrich	1:400,000

Table 2 describes the antibodies used in this manuscript and their dilutions.

Fig.2a Uncropped scans

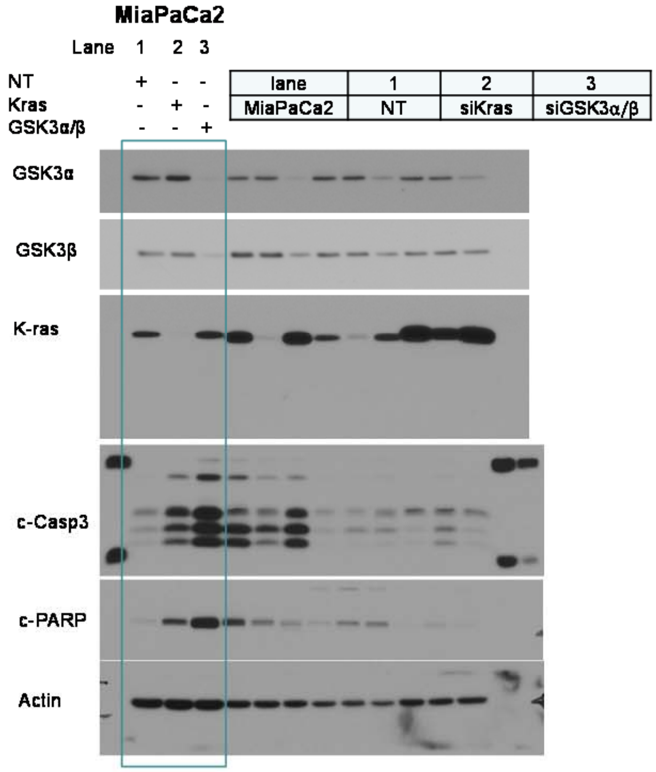

lane	1	2	3
MiaPaCa2	NT	siKras	siGSK3α/β

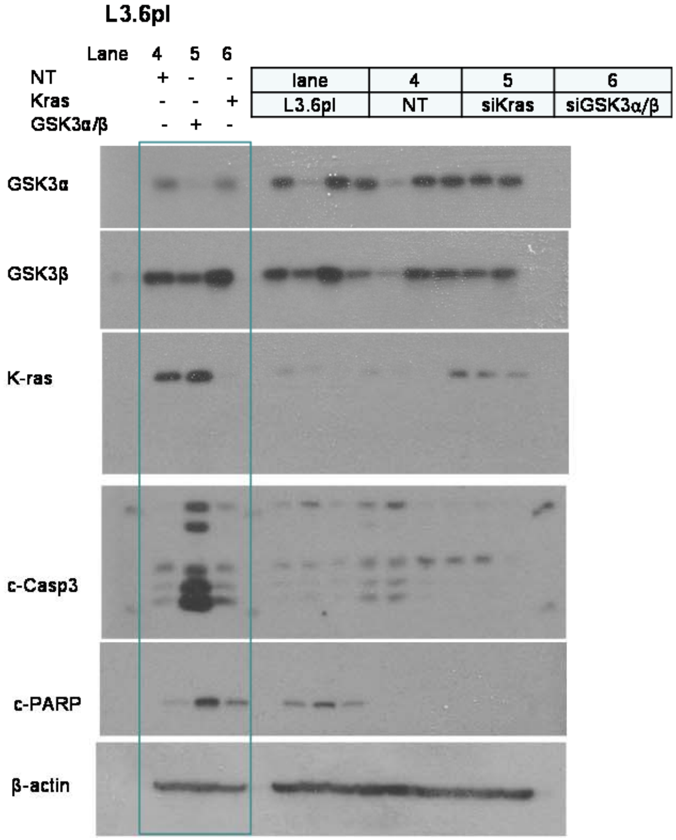

lane	4	5	6
L3.6pl	NT	siKras	siGSK3α/β

Fig.2a Uncropped scans (continued)

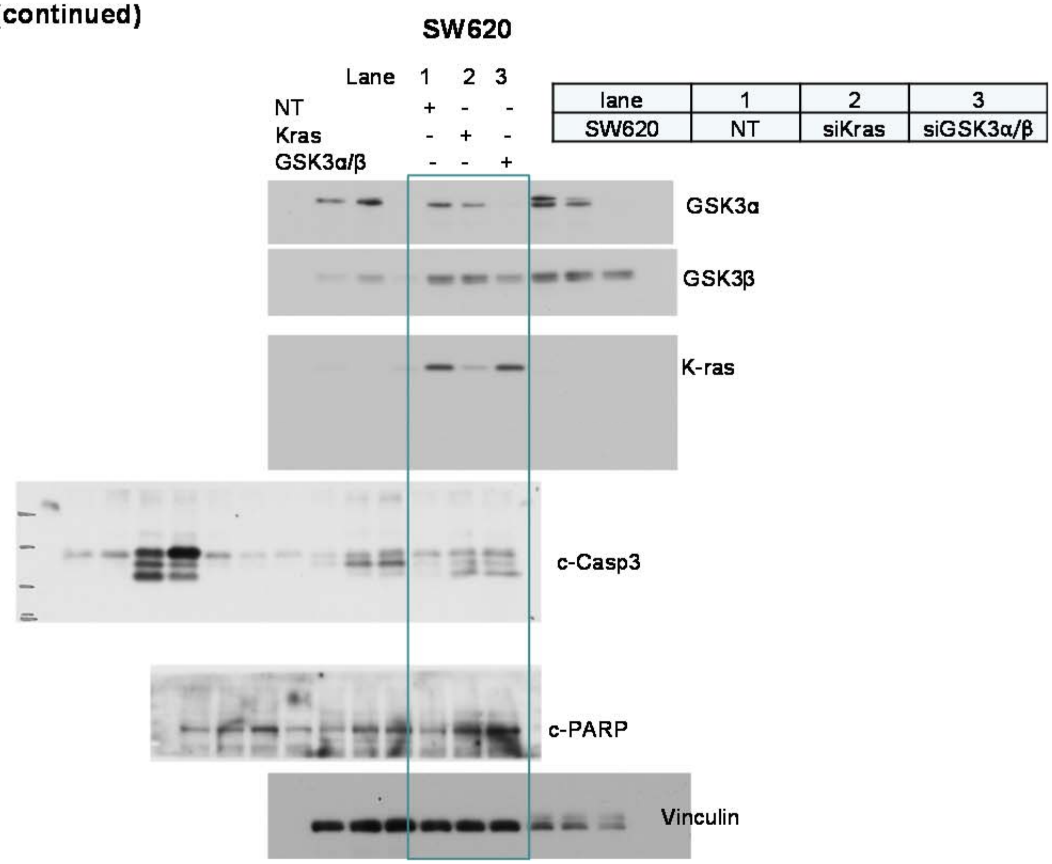

lane	1	2	3
SW620	NT	siKras	siGSK3α/β

Fig.2a Uncropped scans (continued)

Calu6

lane	1	2	3
Calu6	NT	siKras	siGSK3α/β

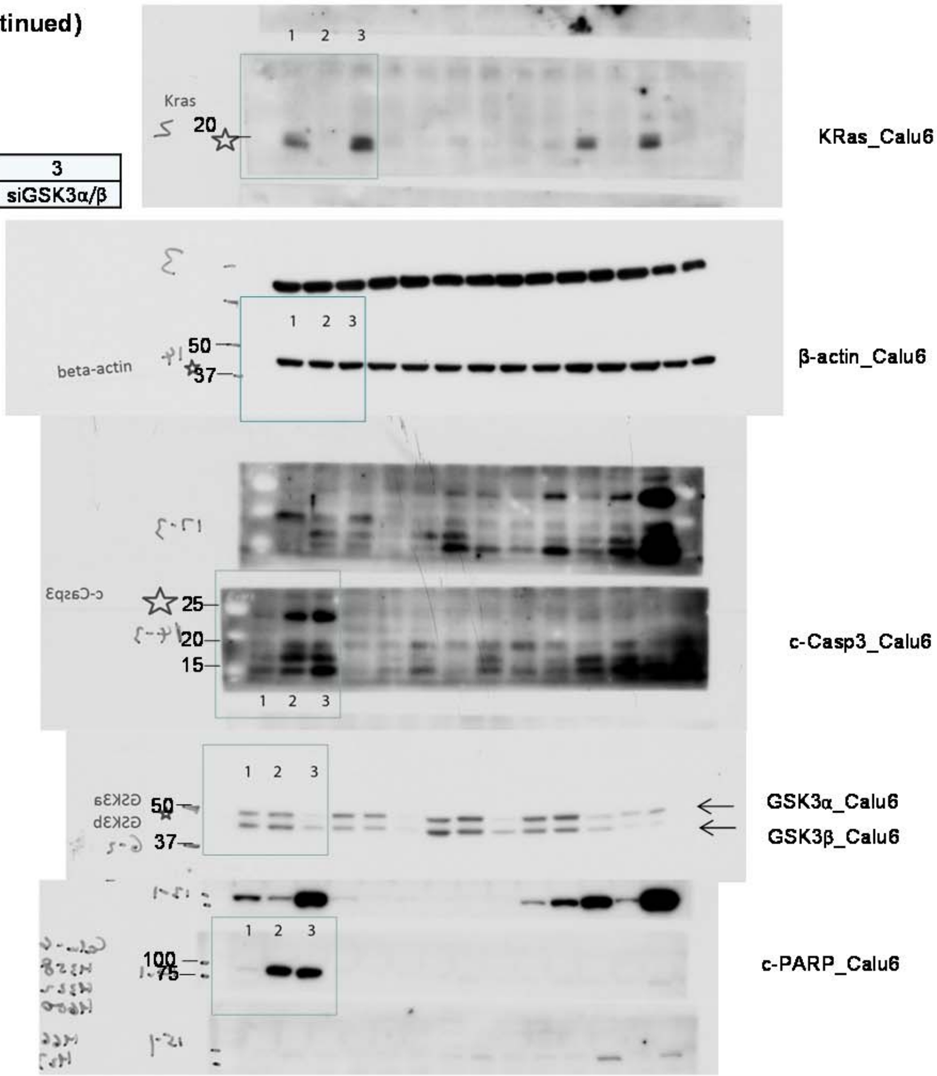

Fig.2a Uncropped scans (continued)

lane	1	2	3
H460	NT	siKras	siGSK3α/β

lane	4	5	6
A549	NT	siKras	siGSK3α/β

lane	7	8	9
DLD1	NT	siKras	siGSK3α/β

lane	10	11	12
HCT8	NT	siKras	siGSK3α/β

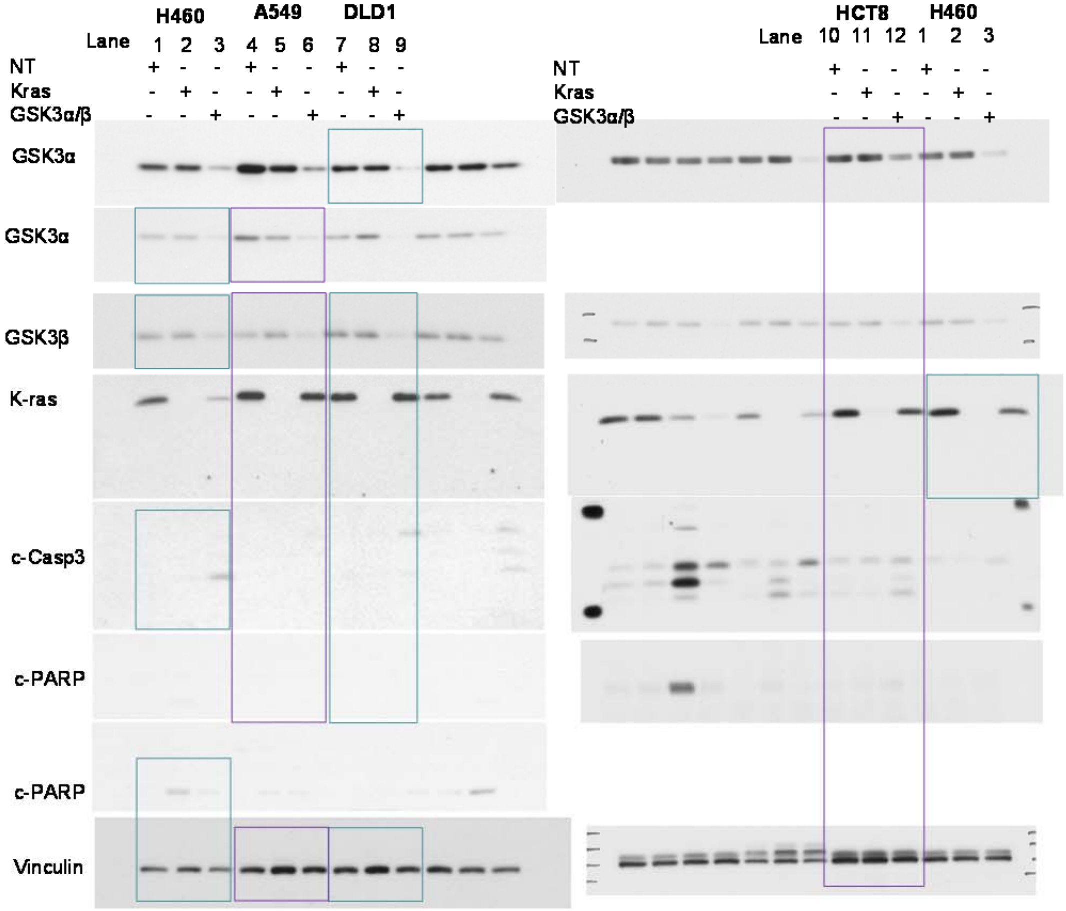

Fig.2b Uncropped scans:

Mt KRas-dependent cells

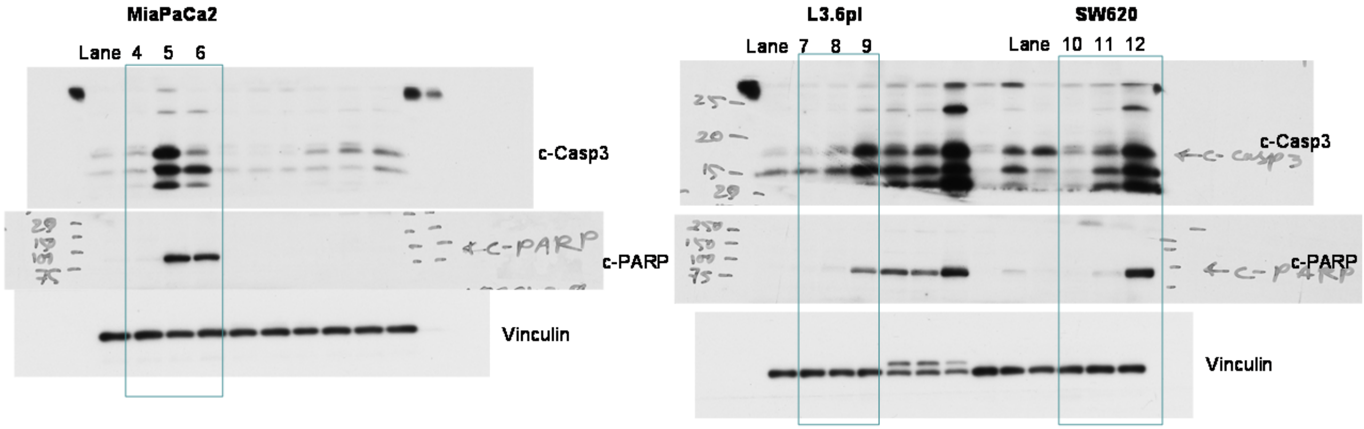

lane	1	2	3
Calu6	SB 0 μM	SB 1 μM	SB 3 μM

lane	4	5	6
MiaPaCa2	SB 0 μM	SB 1 μM	SB 3 μM

lane	7	8	9
L3.6pl	SB 0 μM	SB 1 μM	SB 3 μM

lane	10	11	12
SW620	SB 0 μM	SB 1 μM	SB 3 μM

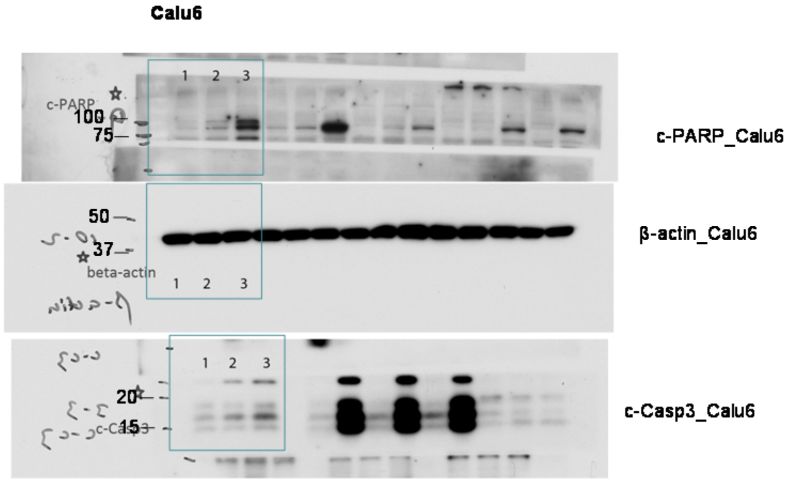

Fig.2b Uncropped scans (continued)

Mt KRas-independent cells

A549

Lane 1 2 3

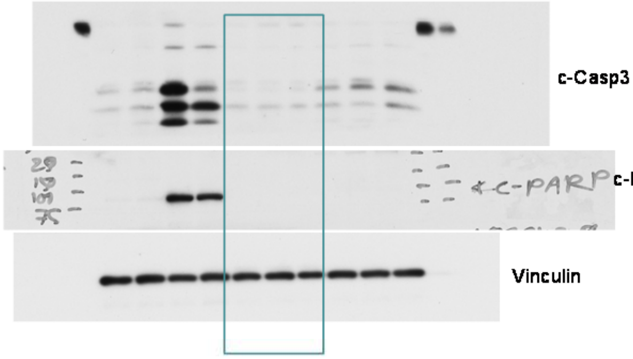

H460

Lane 4 5 6

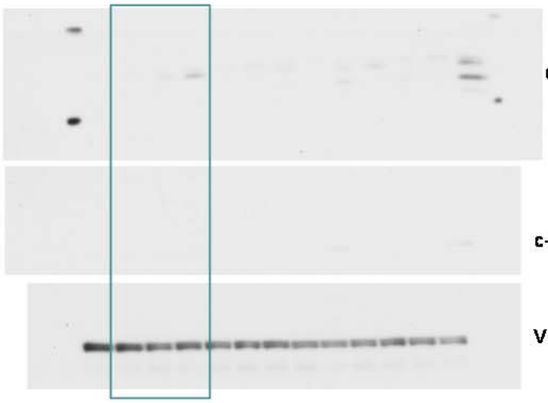

HCT8 **DLD1**

Lane 7 8 9 10 11 12

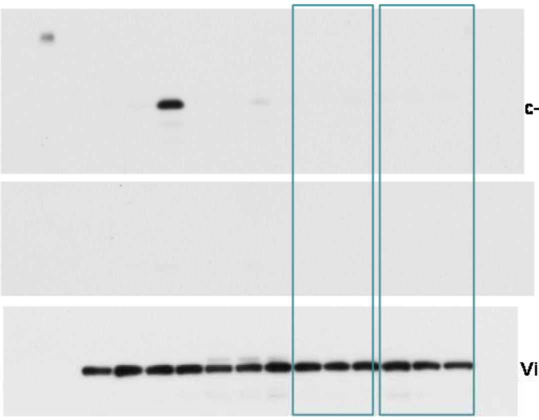

lane	1	2	3
A549	SB 0 μM	SB 1 μM	SB 3 μM

lane	4	5	6
H460	SB 0 μM	SB 1 μM	SB 3 μM

lane	7	8	9
HCT8	SB 0 μM	SB 1 μM	SB 3 μM

lane	10	11	12
DLD1	SB 0 μM	SB 1 μM	SB 3 μM

Fig.5a Uncropped scans:

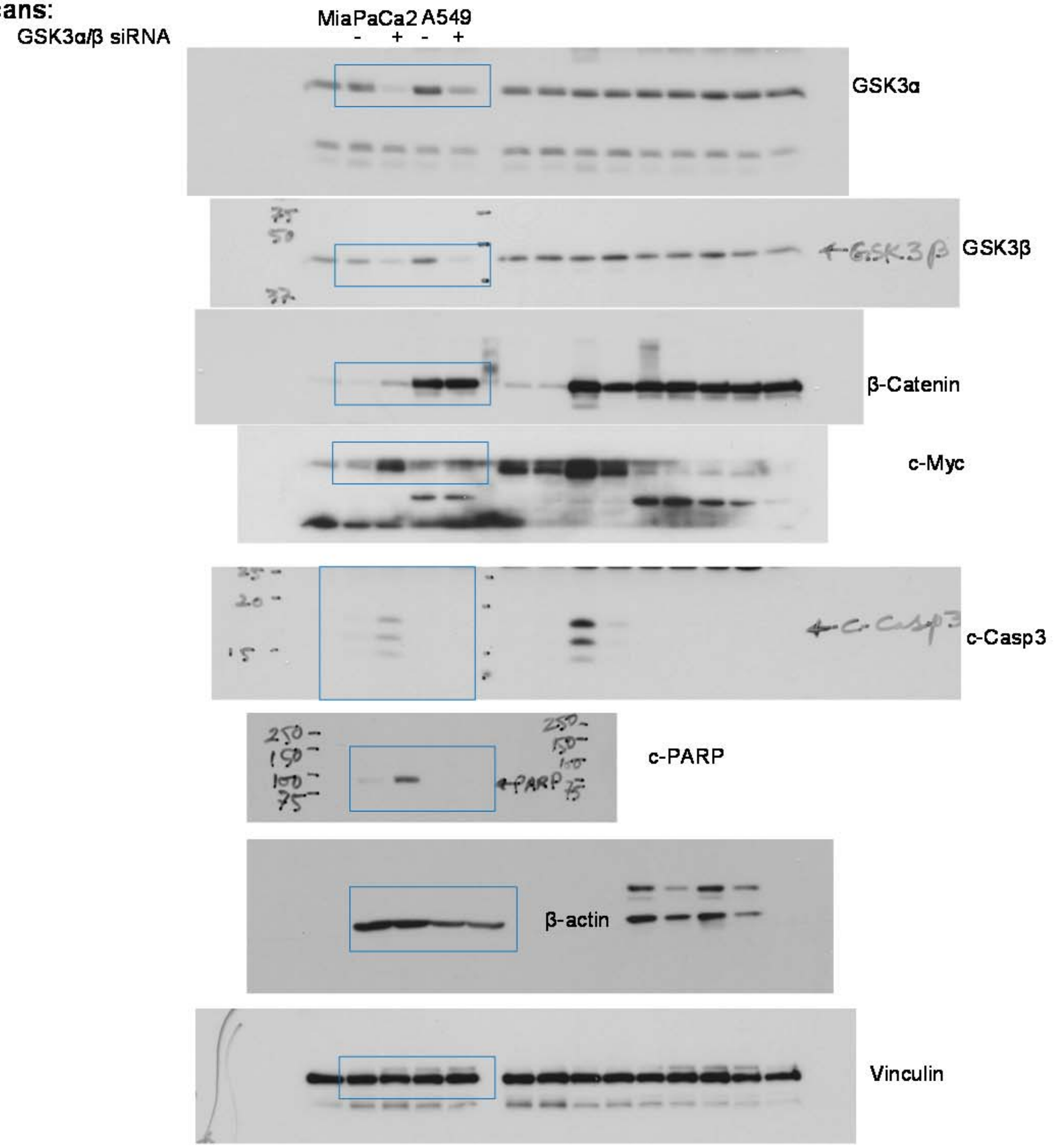

Fig.5b Uncropped scans:

Lane	1	2	3	4	5	6	7	8
Samples	Control	EV	β-Catenin	cMyc T58A	Control	EV	β-Catenin	cMyc T58A
	MiaPaCa2				A549			

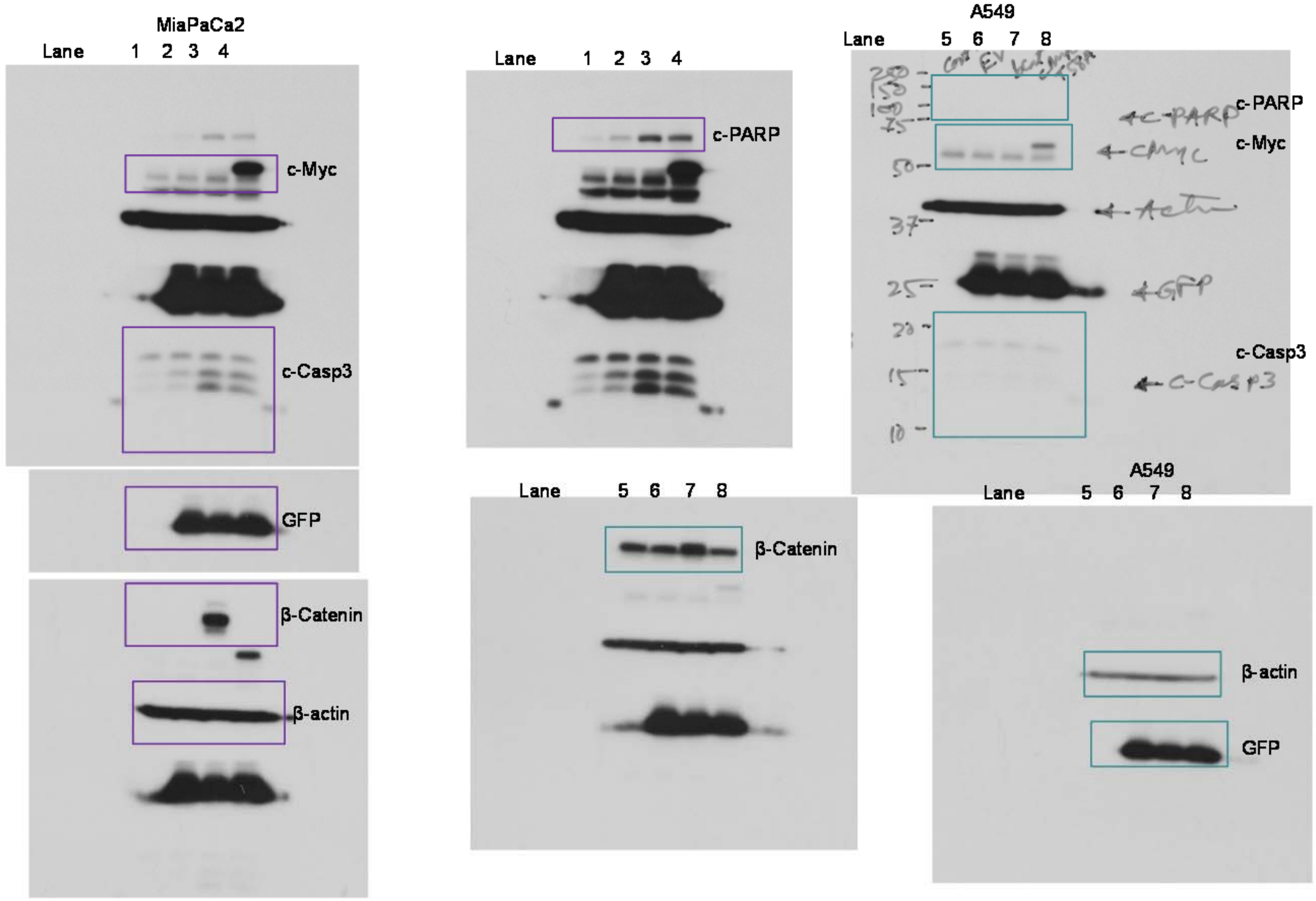

Fig.6a Uncropped scans:

MiaPaCa2

Lane#	1	2	3	4	5	6
SB (μM)	0	1	0	1	0	1
gRNA	SC		C-Myc		β-Cat	

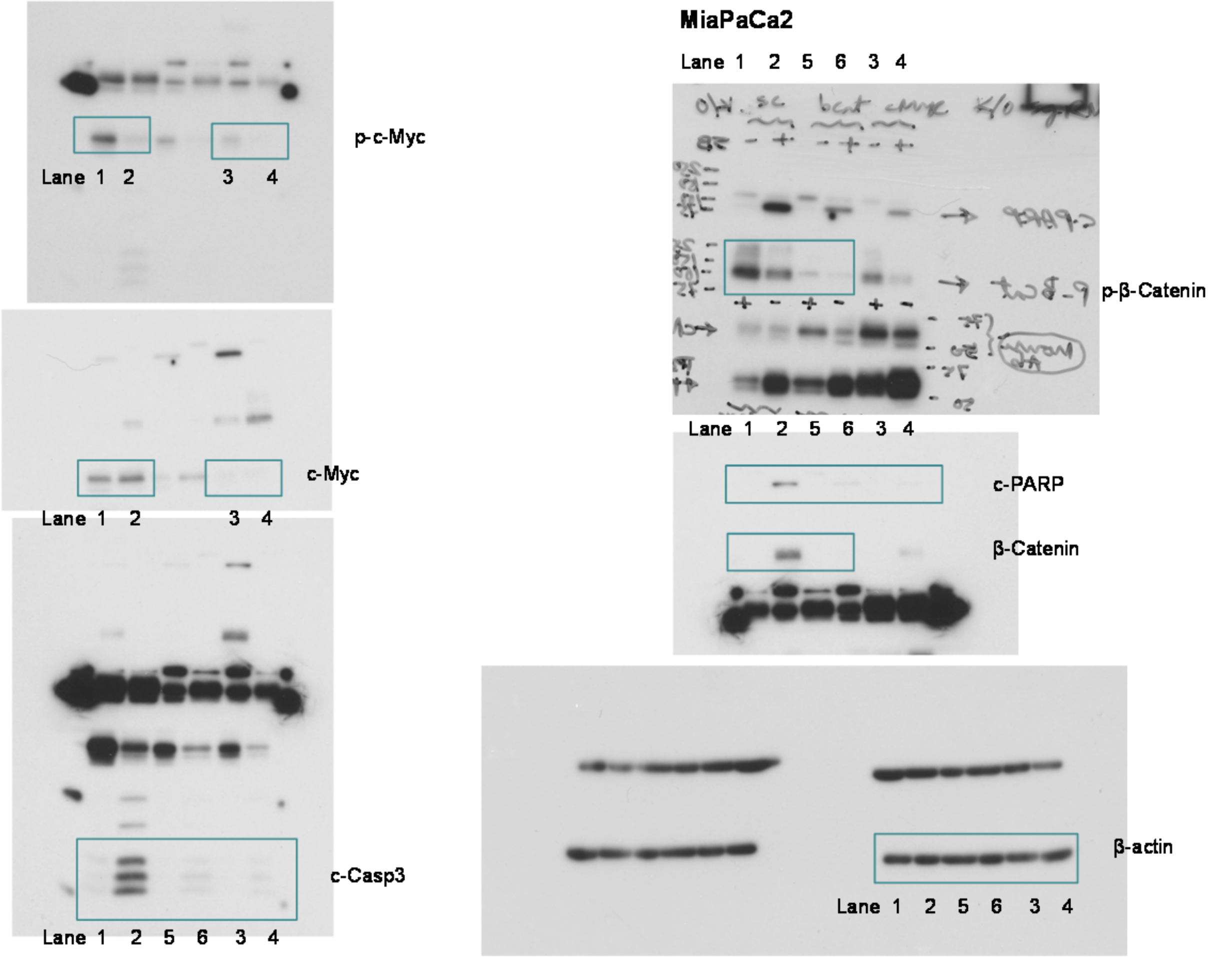

Fig.6b Uncropped scans:

Calu6 (upper panel)

Lane#	1	2	3	4
SB (μM)	0	2	0	2
gRNA	SC		C-Myc	

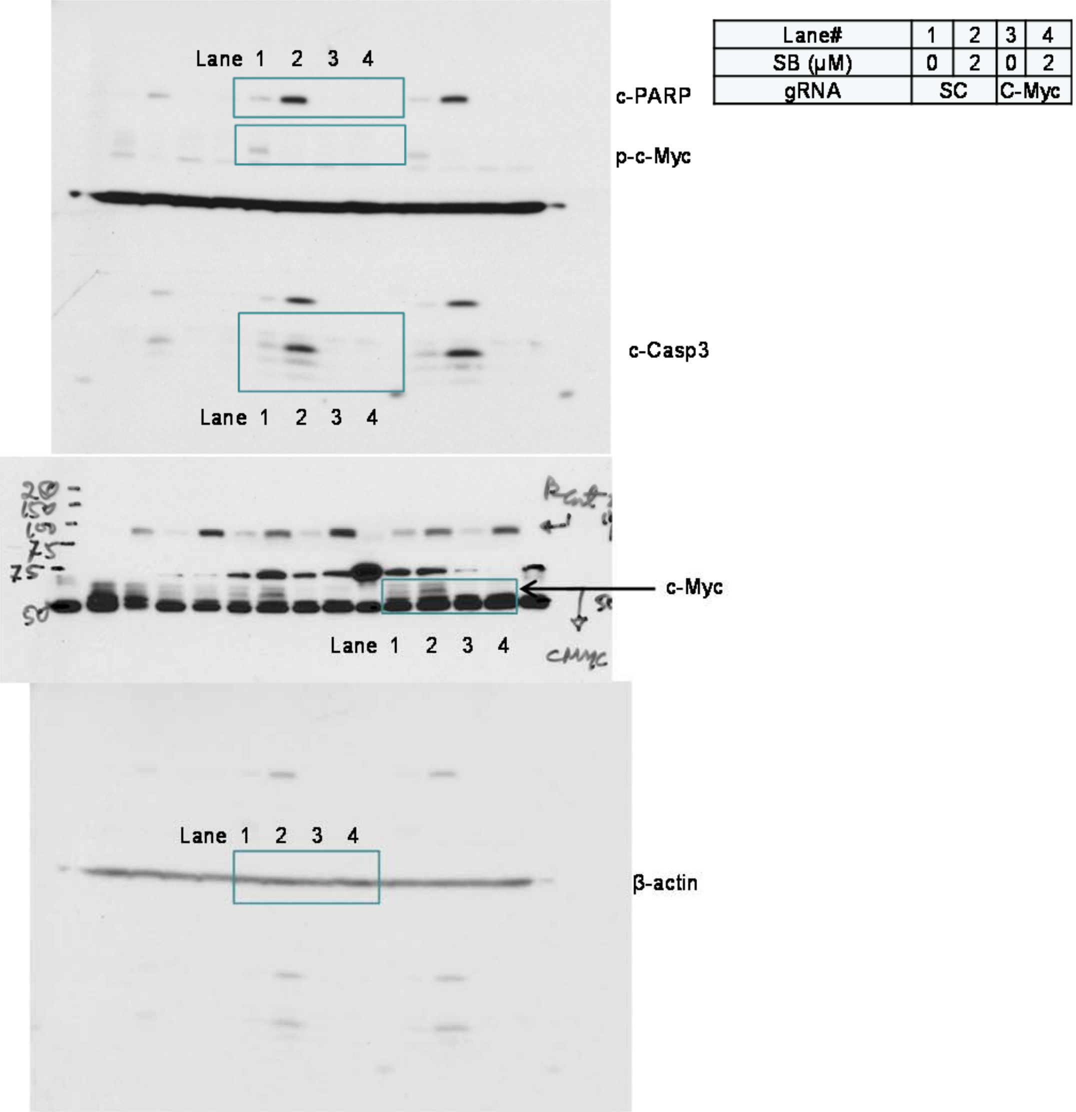

Fig.6b Uncropped scans:

Calu6 (lower panel)

Lane#	1	2	3	4
SB (μM)	0	2	0	2
gRNA	SC		β-catenin	

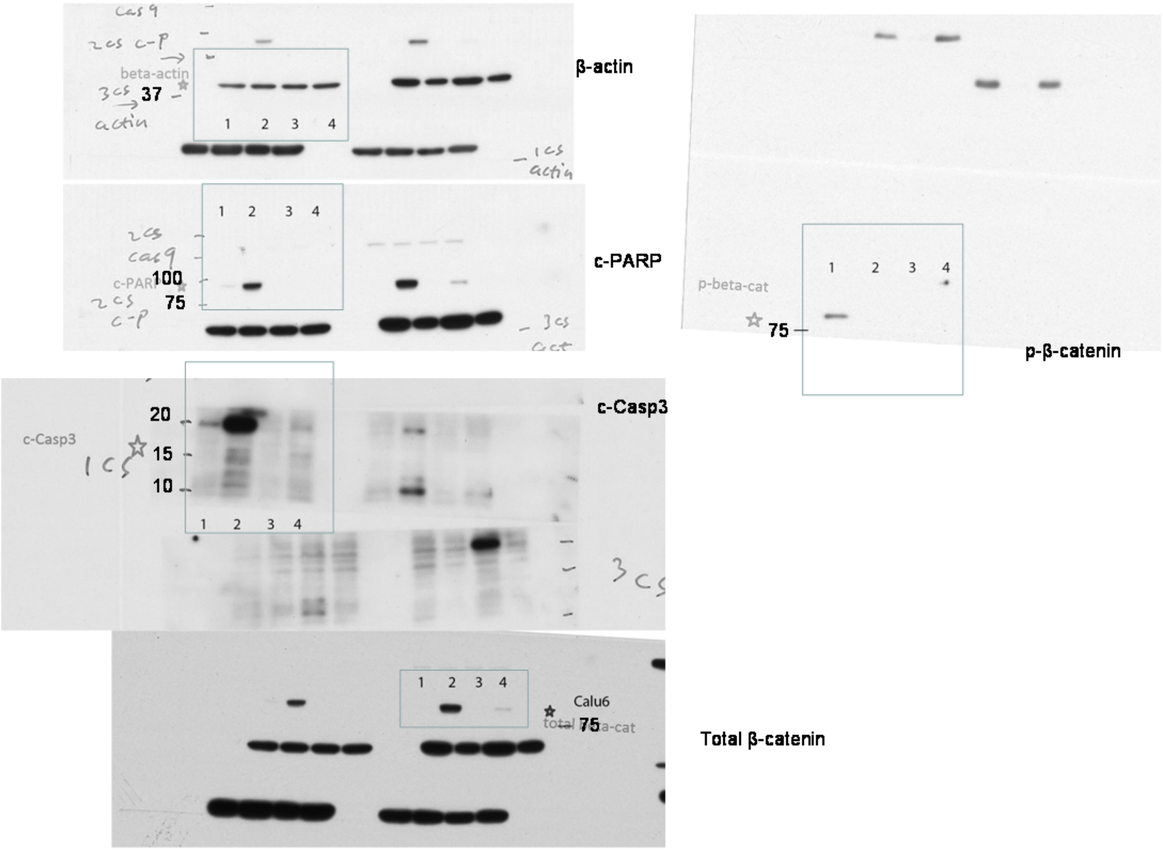

Fig.6c Uncropped scans:

L3.6pl

Lane#	1	2	3	4	5	6
SB (µM)	0	2	0	2	0	2
gRNA	SC		C-Myc		β-Cat	

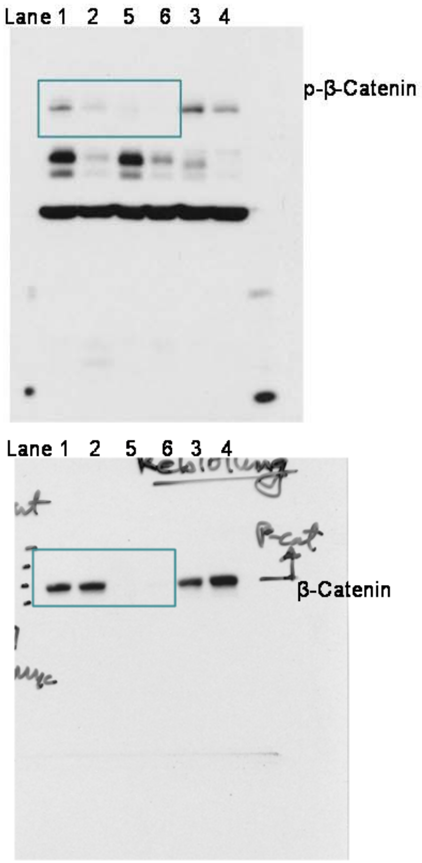

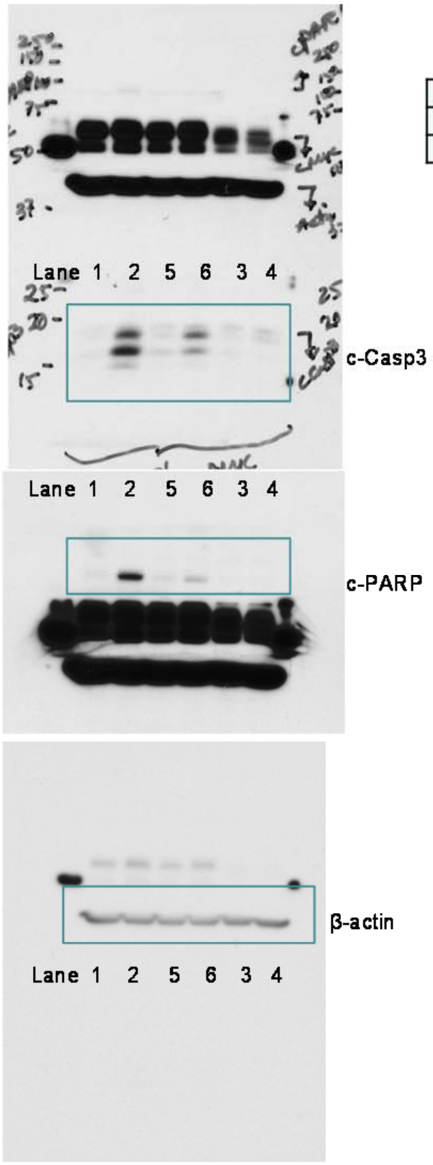

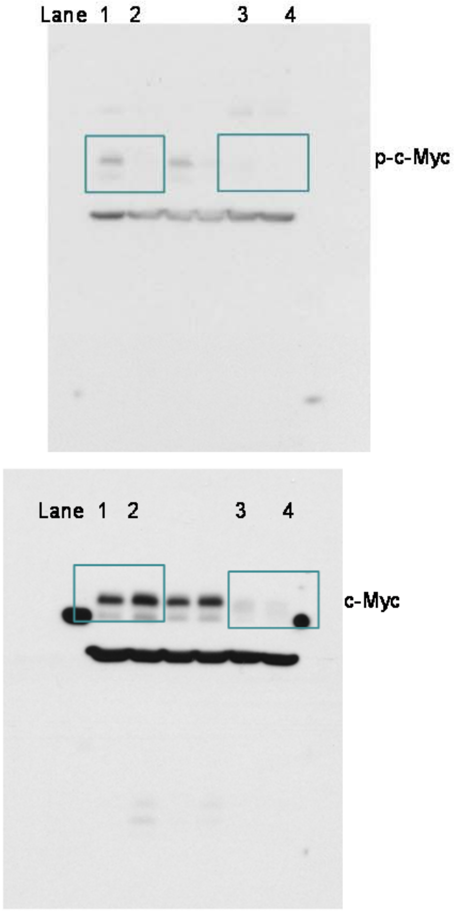

Fig.6d Uncropped scans:

SW620

Lane#	1	2	3	4	5	6
SB (μM)	0	2	0	2	0	2
gRNA	SC		β-Cat		c-Myc	

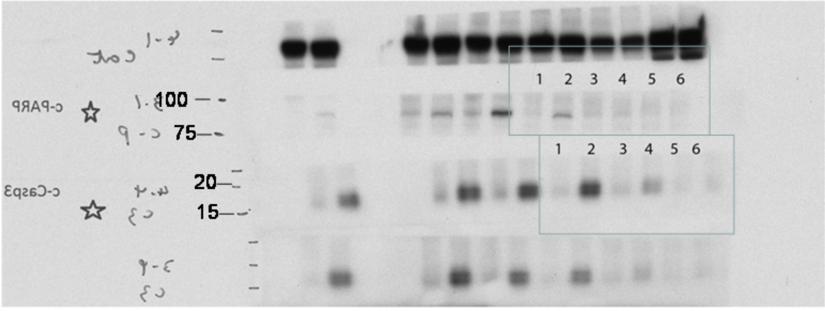

c-PARP_SW620

c-Casp3_SW620

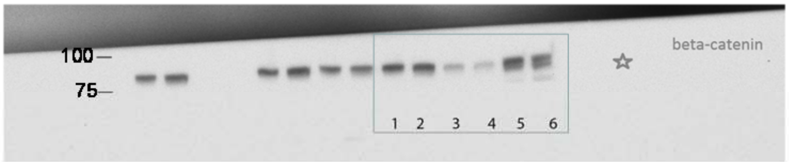

β-catenin _SW620

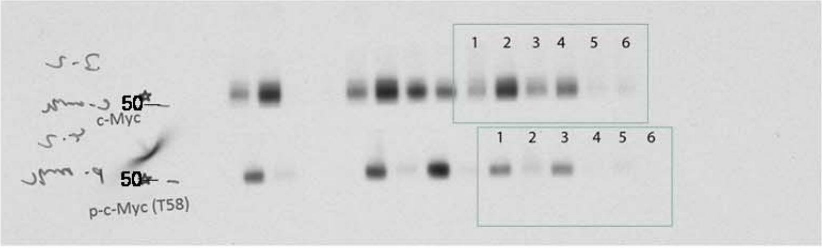

c-Myc _SW620

p-c-Myc (T58)_SW620

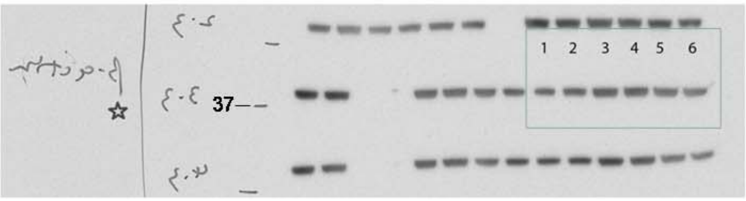

β-actin_SW620

Fig.6d Uncropped scans:

SW620 (continued)

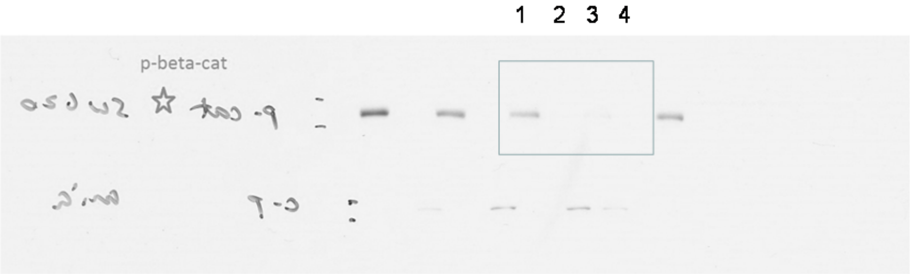

p-β-Catenin_SW620

Fig.6e Uncropped scans:

A549 (upper panel)

Lane#	1	2	3	4
SB (μM)	0	3	0	3
gRNA	SC		C-Myc	

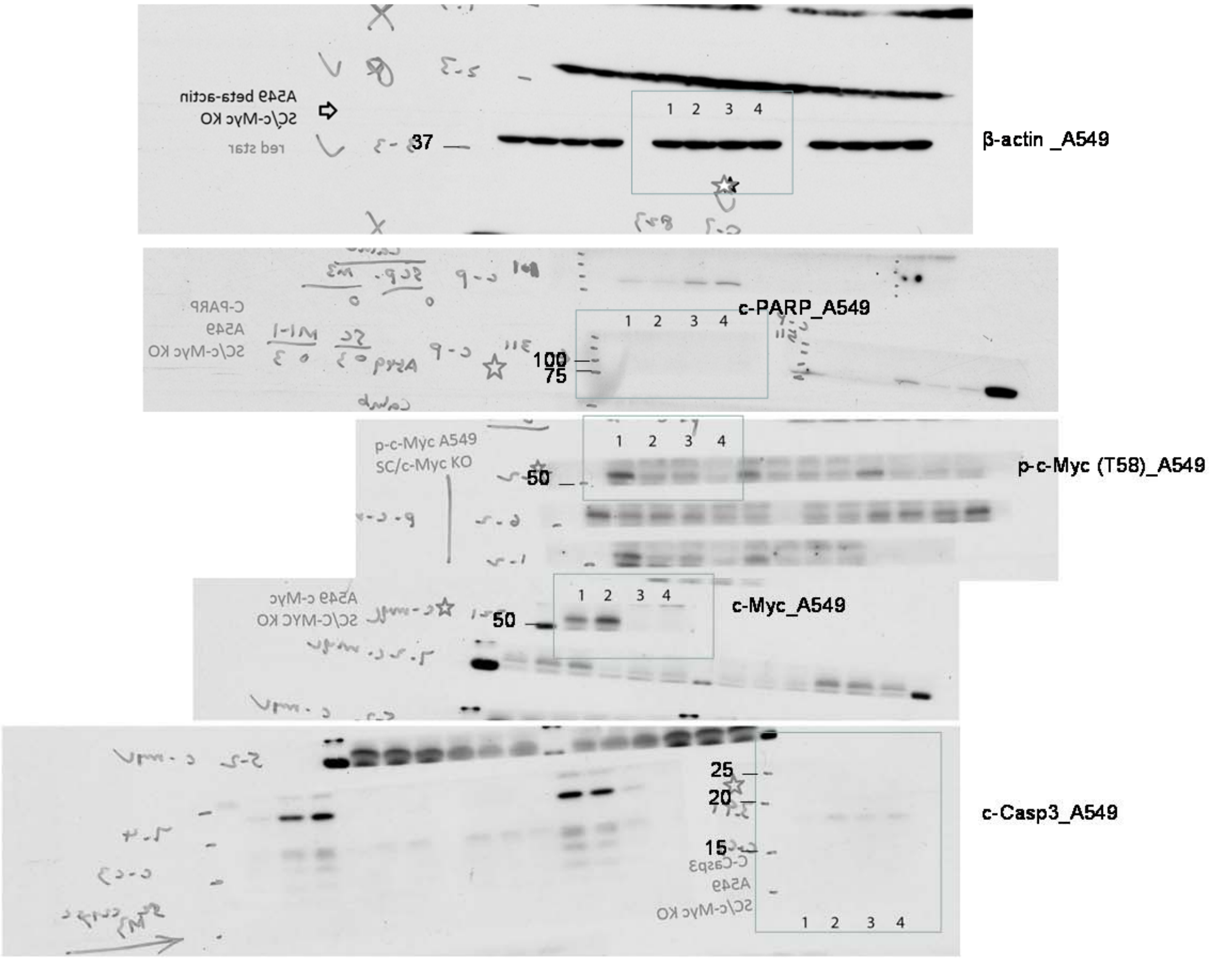

Fig.6e Uncropped scans:

A549 (lower panel)

Lane#	1	2	3	4
SB (μM)	0	3	0	3
gRNA	SC		β-catenin	

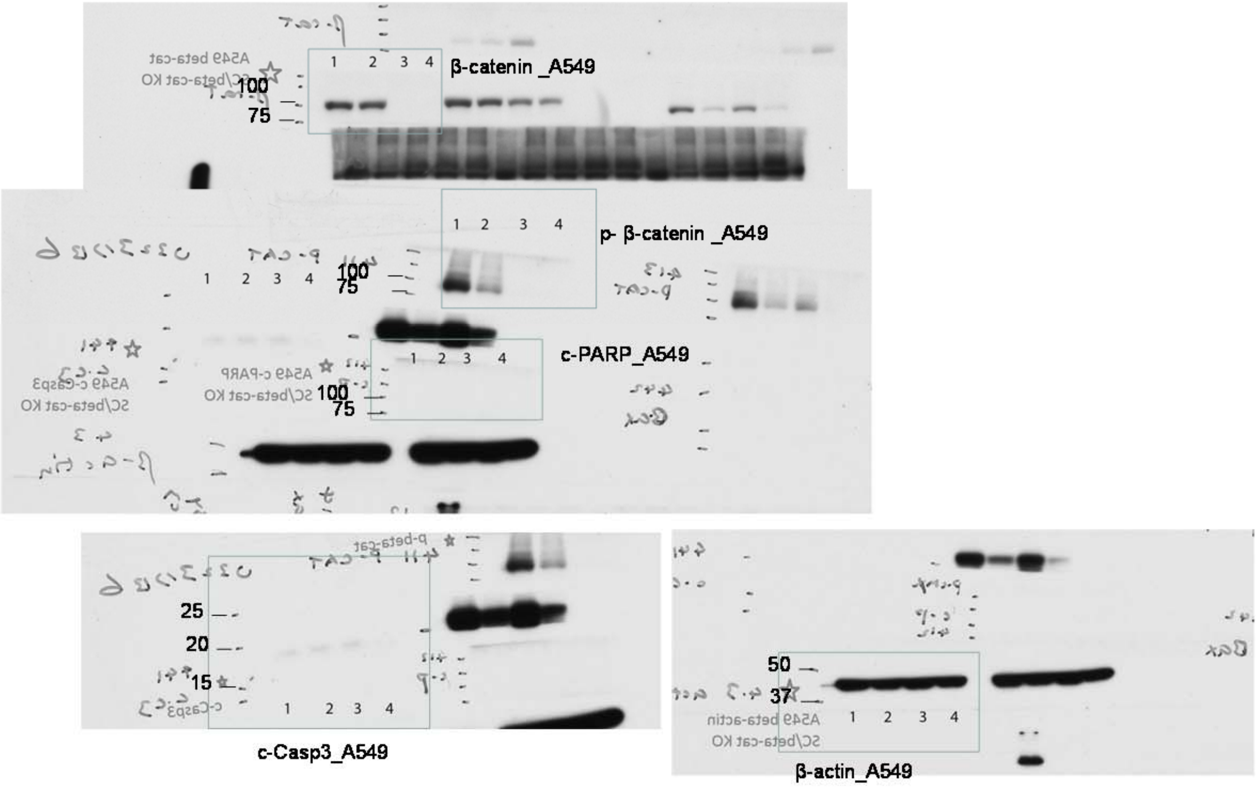

Supplement: Supplementary file 2 — Supplementary Information [file 41467_2018_7644_MOESM2_ESM.pdf]
